# A High-Density SNP Genetic Linkage Map and QTL Analysis of Growth-Related Traits in a Hybrid Family of Oysters (*Crassostrea gigas × Crassostrea angulata*) Using Genotyping-by-Sequencing

**DOI:** 10.1534/g3.116.026971

**Published:** 2016-03-17

**Authors:** Jinpeng Wang, Li Li, Guofan Zhang

**Affiliations:** * Key Laboratory of Experimental Marine Biology, Institute of Oceanology, Chinese Academy of Sciences, Qingdao, Shandong, China 266071; † University of Chinese Academy of Sciences, Beijing, China 100039; ‡ Laboratory for Marine Biology and Biotechnology, Qingdao National Laboratory for Marine Science and Technology, Qingdao, Shandong, China 266071; § National and Local Joint Engineering Laboratory of Ecological Mariculture, Qingdao, Shandong, China 266071; ** Laboratory for Marine Fisheries and Aquaculture, Qingdao National Laboratory for Marine Science and Technology, Qingdao, Shandong, China 266071

**Keywords:** single nucleotide polymorphisms, reduced-representation sequencing, distortion of segregation ratios, missing data imputation, growth-related traits candidate genes

## Abstract

Oysters are among the most important species in global aquaculture. *Crassostrea gigas*, and its subspecies *C. angulata*, are the major cultured species. To determine the genetic basis of growth-related traits in oysters, we constructed a second-generation linkage map from 3367 single-nucleotide polymorphisms (SNPs) based on genotyping-by-sequencing, genotyped from a *C. gigas* × *C. angulata* hybrid family. These 3367 SNPs were distributed on 1695 markers, which were assigned to 10 linkage groups. The genetic linkage map had a total length of 1084.3 cM, with an average of 0.8 cM between markers; it thus represents the densest genetic map constructed for oysters to date. Twenty-seven quantitative trait loci (QTL) for five growth-related traits were detected. These QTL could explain 4.2–7.7% (mean = 5.4%) of the phenotypic variation. In total, 50.8% of phenotypic variance for shell width, 7.7% for mass weight, and 34.1% for soft tissue weight were explained. The detected QTL were distributed among eight linkage groups, and more than half (16) were concentrated within narrow regions in their respective linkage groups. Thirty-eight annotated genes were identified within the QTL regions, two of which are key genes for carbohydrate metabolism. Other genes were found to participate in assembly and regulation of the actin cytoskeleton, signal transduction, and regulation of cell differentiation and development. The newly developed high-density genetic map, and the QTL and candidate genes identified provide a valuable genetic resource and a basis for marker-assisted selection for *C. gigas* and *C. angulata*.

Originating in East Asia, the Pacific oyster (*Crassostrea gigas*) has been introduced to many countries around the globe, and has become an important species in aquaculture in many countries including the USA, Canada, Australia, France, and South Africa, due to its rapid growth rate and high productivity (Guo 2009). Of the many cultured aquatic animals, the worldwide production was highest for *Crassostrea gigas* for many decades; in 2013, global production was 555,994 metric tons, with an estimated value of approximately US$ 1.3 billion (Food and Agriculture Organization 2015). In China, it is a dominant species in the intertidal zone, and a major cultured species on the northern coast. In contrast, *C. angulata*, a subspecies of *C. gigas* (Wang *et al.* 2010), is widely distributed and cultured on the southern coast of China. The two subspecies present differentiation in growth rate when cultured in the same environment (L. Li *et al.*, unpublished data) and the similarity of genic regions of them exceed 90% (H. G. Qi *et al.*, unpublished data). The two subspecies can hybridize, and the hybrids are fertile (Huvet *et al.* 2002; Patrick *et al.* 2002; Leitão *et al.* 2007). Farming of both *C. gigas* and *C. angulata* is confronted with problems, including a decline in growth rate, germplasm degeneration, and summer mortality (Le Roux *et al.* 2002; Buestel *et al.* 2009; Cotter *et al.* 2010). Several traditional selective breeding programs have been launched to increase growth rate and decrease summer mortality (Ward *et al.* 2000; Degremont *et al.* 2010; Li *et al.* 2011; Wang *et al.* 2012b). However, traditional selection is time-consuming and labor-intensive. Marker-assisted selection (MAS) is becoming popular in crop and aquaculture breeding programs for its high efficiency. Construction of a linkage map and mapping of trait-related quantitative trait loci (QTL) is required as a foundation for implementation of MAS.

Several linkage maps have been constructed for the Pacific oyster using amplified fragment-length polymorphism (AFLP) markers, or combinations of AFLP and microsatellite markers (Li and Guo 2004; Guo *et al.* 2012), microsatellite DNA markers (Hubert and Hedgecock 2004; Hubert *et al.* 2009; Plough and Hedgecock 2011), and a combination of microsatellite markers and single-nucleotide polymorphisms (SNPs) (Sauvage *et al.* 2010; Zhong *et al.* 2014). However, only a limited number of QTL have been identified for economic traits, such as five QTL peaks for oyster weight on four chromosomes (Hedgecock *et al.* 2007), three QTL for growth-related quantitative traits (Guo *et al.* 2012), and two QTL for glycogen content (Zhong *et al.* 2014). However, these linkage maps were constructed mainly based on low-density microsatellite DNA markers and AFLP markers, which limited the resolution for QTL mapping. Recently, Hedgecock *et al.* (2015) constructed higher-density, second-generation linkage maps for the Pacific oyster based on 1085 coding SNPs generated by expressed sequence tag (EST) sequencing, as well as 66 microsatellite DNA markers, in five mapping families with an average total map length of 588 cM, a mean of 584 markers per map, and an average marker spacing of 1.04 cM.

Compared with micro-array based and PCR-based SNP genotyping methods, such as high-resolution melting (HRM) (Wang *et al.* 2015), reduced representation library (RRL) sequencing is a more economical and efficient way to develop abundant SNP markers for construction of a dense linkage map (Van Tassell *et al.* 2008). Popular RRL methods include restriction site-associated DNA sequencing (RAD) (Baird *et al.* 2008) and genotyping-by-sequencing (GBS) (Elshire *et al.* 2011), which utilize restriction endonucleases to generate DNA fragments flanking the restriction sites of these enzymes. These methods are widely used in model and nonmodel species, including marine bivalve species, such as *Pinctada fucata* (Li and He 2014) and *Chlamys farreri* (Jiao *et al.* 2014).

To make full use of interspecies variations, a number of hybrid families of *C. gigas* and *C. angulata* were constructed to dissect the genetic basis for the economical traits, and to accelerate molecular breeding. In the present study, one hybrid family was used to screen the GBS-based SNPs. A high-density genetic map containing a number of GBS-based SNPs was constructed, QTL associated with growth-related traits were detected, and candidate genes for growth-related traits were identified using the oyster reference genome (Zhang *et al.* 2012). Considering the high similarity of genic regions of *C. gigas* and *C. angulata*, we intend to detect QTL and candidate genes that have high chance of being used in both these subspecies. These data will help to promote genetic dissection of important economic traits and subsequent MAS for oysters.

## Materials and Methods

### Ethics statement

No specific permissions were required to conduct experiments with Pacific oyster in China. All oysters used in this study were cultured in a fishery farm and cannot cause environmental harm.

### Mapping family and measurement of growth-related traits

The mapping family was constructed in a hatchery in Laoshan, Qingdao, China (N36°12′, E120°41′) in the summer of 2012. The male parent was a 2-yr-old hybrid of *C. gigas* and *C. angulata* produced and cultured in Nan’ao Island, Shantou, China (N23°25′, E117°01′). The female parent was a 2-yr-old wild *C. gigas* individual from Jiaonan, Qingdao, China (N35°44′, E119°56′). The mating of these two parents generated an F1 cross. Both the parent oysters were dissected manually, and their gonads were rinsed into 1 liter of seawater. The gill tissues of the two parent oysters were sampled and stored in 70% alcohol for DNA extraction. The sperm and eggs were activated separately in seawater for 30 min and 1 hr before fertilization, respectively. The larval progeny were reared in a 70-liter barrel in the hatchery, for 1 month, until all individuals adhered to the adhesion substrate. The progeny were then cultured on a farm located in Jiaonan, Qingdao. When the progeny were 14 months old, the shell height, shell length, shell width, gross weight, and soft tissue weight of each of the 106 progeny were measured. Correlations between these traits were evaluated by Pearson’s correlation coefficient (Anthony *et al.* 2007) using the *cor* statistics function of R (R Core Team 2008). For each offspring, gender was determined, and the gill tissue was sampled and frozen in liquid nitrogen for subsequent DNA extraction. Student’s *t*-test was used to compare the five growth-related traits between female and male progeny.

### Genotyping-by-sequencing

DNA was extracted from the parent oysters using a standard phenol/chloroform extraction method (Green and Sambrook 2012). Following extraction, the DNA was examined by electrophoresis to confirm its integrity. The DNA concentration was quantified using Qubit 2.0 (Thermo Fisher Scientific, Waltham, MA). The Perl package RestrictionDigest (Wang *et al.* 2016) was used to determine the appropriate enzyme combination for digesting the genomic DNA and the *Eco*RI–*Hin*fI combination was selected. GBS sequencing libraries were constructed according to Poland *et al.* (2012), with slight modifications. First, 200 ng of DNA from each oyster was double-digested with *Eco*RI [New England Biolabs (NEB), Ipswich, MA] and *Hin*fI (NEB) in CutSmart buffer (NEB) at 37° for 2 hr. Second, the digested DNA fragments were ligated to two kinds of adaptors using T4 DNA ligase (NEB) in T4 ligase buffer: adaptor 1 ends with an overhang matching that produced by *Eco*RI, and adaptor 2 ends with an overhang matching that produced by *Hin*fI. The adaptors were quantified using Qubit 2.0 to equalize the adaptors used for every individual. Nineteen barcodes, 4–9 bp in length, located within adaptor 1 were designed to distinguish different individuals. Third, DNA fragments from the second step with different barcodes were pooled, and DNA fragments from the two parents and the 106 progeny were mixed to generate six pools. Fourth, the pooled samples were separated on a 2% agarose gel, and DNA fragments of 400–600 bp (including adaptors) were purified using a QIAquick Gel Extraction Kit (Qiagen, Hilden, Germany). Fifth, the selected DNA fragments were amplified by PCR using NEBNext High-Fidelity 2 × PCR Master Mix (NEB) with the following procedure: 95° for 30 sec, 62° for 30 sec, and 68° for 30 sec for 16 cycles. Sixth, the amplified products were purified using Agencourt AMPure XP beads (Beckman Coulter, High Wycombe, UK). Single-end 100 bp (100SE) sequencing of the purified DNA fragments, in the region adjacent to adaptor 1, was performed on the Illumina HiSequation 2500 system (Illumina, San Diego, CA) in accordance with the manufacturer’s recommendations.

The Stacks software was used to analyze the sequencing data and to genotype DNA variations in the mapping family (Catchen *et al.* 2013). First, raw reads were allocated to each individual by running “process_radtags” in Stacks; during this procedure, reads with incorrect barcodes or low quality were discarded, to guarantee the read quality. Second, reads of each individual were aligned to the Pacific oyster reference genome oyster_v9 (GenBank accession no. GCA_000297895.1) by using BWA (Li and Durbin 2009) with parameter “aln –n 2 –M 3;” the alignment result files in Sequence Alignment/Map (SAM) (Li *et al.* 2009) format were used for subsequent analysis. To decrease the negative impact of repetitive sequences on genotyping, uniquely aligned reads were extracted by run the bash command “grep -v ’XT:A:R’ ” for subsequent analysis. Third, “ref_map.pl” in Stacks was used to genotype markers. The maximum-likelihood SNP model was used to call genotypes and the genotype likelihood ratio test significance level was set as 0.05. The minimum depth of coverage to report a stack was set as 3. The map type was set as “CP.” The output file type of genotypes was set as “joinmap.” Custom Perl scripts were used to filter output.

### Linkage map construction

The whole RAD locus was used as a single marker in map construction. Linkage map construction was performed according to the strategy developed by Ward *et al.* (2013). First, maternal and paternal linkage maps were constructed separately using JoinMap 4.0 (Van Ooijen 2006). Markers in the lm × ll segregation type (the female parent is heterozygous, and the male parent is homozygous) were used to construct the female linkage map, and markers in the nn × np segregation type (the female parent is homozygous, and the male parent is heterozygous) were used to construct the male linkage map. For construction of each sex-specific linkage map, markers were grouped into linkage groups under the cross-pollination (CP) population type. The threshold independence logarithm of the odds (LOD) score was set to 5.0–7.0 for the female map, and 5.0–9.0 for the male map. Then markers were initially ordered along the linkage groups using the maximum likelihood algorithm of JoinMap 4.0 for each sex-specific linkage map. Once the markers were given an initial order along the linkage groups, imputation of missing data and genotyping errors was performed using Maskov (Ward *et al.* 2013). The imputation parameters in Maskov were set to E = 3, and the maximum amount of missing data were set at 40%. After the imputation, imputed markers were again input into JoinMap 4.0, and the map distances were calculated using Kosambi’s mapping function (Kosambi 1943). Then, the imputed markers from the sex-specific maps were used to construct the sex-average linkage map. The LOD score was set to 6.0–9.0. Map distances were calculated using Kosambi’s mapping function. Observed genome length (Goa) was taken as the total length of all markers on each linkage group. Two methods were used to estimate the expected genome length (Ge) of the linkage map: (1) Ge1 was calculated by multiplying the length of each genetic linkage group by (m + 1)/(m – 1), where m is the number of loci in each genetic linkage group (Chakravarti *et al.* 1991). (2) Ge2 was calculated by adding 2s (s is the average interval of the genetic map) to the length of each linkage group, to account for chromosome ends (Fishman *et al.* 2001). The average of Ge1 and Ge2 was used as Ge. Genome coverage (Coa) was denoted by Goa/Ge. For each linkage group, the average spacing of markers was calculated by taking the mean value of all interval distances between adjacent markers. For the whole linkage map, the average spacing of markers was calculated by taking the mean value of all linkage groups’ average spacing of markers.

### QTL detection

Prior to QTL mapping, growth traits were tested to determine whether they were normally distributed via the Shapiro–Wilk test (Royston 1982) using R. If the trait did not show a normal distribution, the values were transformed to show a normal distribution by power transformation. MapQTL 5.0 (Van Ooijen 2004) was used to map growth-related QTL. The interval mapping and multi-QTL mapping (MQM) methods were used to map QTL. For each trait, the interval mapping method was first used to reveal general information on candidate QTL. Then, the MQM method was used for every linkage group, under the condition that the loci with the largest LOD scores in the other linkage groups were treated as cofactors. The LOD scores in each linkage group were fixed after the cofactors in all other linkage groups had been searched. Permutations were executed to determine the genome-wide significance threshold of the LOD scores (*P* < 0.05), with the number of permutations set at 10,000. LOD peaks bigger than LOD threshold were identified as QTL. The location of each QTL was determined according to the location of its LOD peak and the surrounding region. Candidate genes for growth-related traits were identified by mapping the corresponding markers in QTL regions to the reference genome, followed by retrieval of the corresponding gene ID from the gene annotation file.

### Data availability

Supplemental Material, File S1 contains histograms for the five growth-related traits. File S2 contains boxplots of comparisons for the five growth-related traits between female and male progeny. File S3 contains charts of the 10 linkage groups on the sex-average map. File S4 contains charts of the distribution of distorted markers on the 10 linkage groups on the sex-average map. Table S1 contains values of five growth-related traits and gender for every progeny of the mapping family. Table S2 contains an analysis of correlations between the five growth-related traits in the mapping family. Table S3 contains the details of the sequencing and genotyping results of the six genotyping-by-sequencing libraries of the mapping family. Table S4 contains a summary of SNPs identified in the mapping family. Table S5 contains the genotypes of the 1695 markers mapped. Table S6 contains the sequences of the mapped markers. Table S7 contains the markers and their positions on each of the 10 linkage groups on the sex-average map. Table S8 contains the comparison of genetic distances and expected recombination counts before and after the imputation for the sex-specific maps, by linkage group. Table S9 contains the lengths and numbers of the markers for the sex-specific and the sex-average maps, by linkage group. Table S10 contains the proportion of distorted markers for each linkage group on the sex-average map. Table S11 contains the IDs and symbols for the annotated genes in the QTL regions.

## Results

### Phenotyping of growth-related traits

Shell height ranged from 3.7 cm to 9.9 cm, with an average of 7.6 cm (Table S1). Shell length ranged from 2.6 cm to 5.8 cm, with an average of 4.5 cm. Shell width ranged from 1.8 cm to 3.2 cm, with an average of 2.4 cm. Mass weight ranged from 1 g to 7.6 g, with an average of 4.6 g. Soft tissue weight ranged from 0.2 g to 1.9 g, with an average of 1 g. Pearson correlation coefficients between growth-related traits ranged from 0.397 to 0.852 (Table S2). Mass weight and soft tissue weight showed the highest correlation (0.852), and shell height and shell width showed the lowest correlation (0.397). Not all growth-related traits showed significant correlations; hence, they were not integrated into a single composite trait, but were analyzed separately for QTL mapping. Values for shell length, shell width, mass weight, and soft tissue weight followed a normal distribution, but values for shell height did not (File S1). The shell height values were transformed for normality by taking the exponential: *y* = exp(*x*^2/1000), where *x* represents trait values before transformation and *y* represents trait values after transformation. Gender affected growth-related traits in this mapping population (File S2). Female progeny showed significantly larger values for five growth-related traits than male progeny. Male progeny showed larger variance for five growth-related traits than female progeny; this indicated that gender should be treated as a covariant when mapping QTL.

### Genotyping-by-sequencing

Digestions of the Pacific oyster reference genome with different enzyme combinations were simulated with RestrictionDigest, and *Eco*RI–*Hin*fI was selected to digest the genomic DNA for library preparation. Simulated *Eco*RI–*Hin*fI digestion revealed that the digested fragments covered 6.84 Mb of the genome, 48.9% of which were located in intergenic regions, and 51.1% in genic regions, with 43.3% in introns and 7.8% in exons. This simulated distribution was concordant with the genomic region proportions found in the reference genome (Zhang *et al.* 2012), indicating that digestion with *Eco*RI–*Hin*fI would generate an even distribution of digested fragments across the genome. To identify genomic variation in the mapping population, *Eco*RI–*Hin*fI RRLs were constructed. To ensure sufficient read depth at potential variation loci, deep sequencing of both parents and all progeny was performed. Details of the sequencing and genotyping results are listed in Table S3. In summary, 100SE sequencing of six GBS libraries generated 664.3 M of raw reads with a GC content of 43%. After filtering the low-quality reads, 473.9 M (71.3%) clean reads remained. Cleaned reads were mapped against the Pacific oyster reference genome with BWA. The total mapped base lengths covered by the ∼90-bp short reads from the parental *Eco*RI–*Hin*fI libraries was approximately 6.58 Mb, representing 1.2% of the published reference genome sequence. Of the 6.58 Mb mapped, 47.5% were located in intergenic regions, and 52.5% were located in genic regions, with 38.2% located in introns and 14.3% located in exons. The GC content of the mapped regions was 36.2% for intergenic regions, 39% for genic regions, 35.2% for introns, and 49% for exons.

In total, the Stacks software generated 65,031 RAD loci in the mapping family, of which 15,783 (24.3%) were polymorphic. These polymorphic RAD loci contained 40,445 SNPs, with an average of 2.6 SNPs per locus. Relative to the reference genome, we identified a total of 40,445 single nucleotide substitutions, among which transitions (A ↔ G or C ↔ T) accounted for 48.1%, and transversions (A ↔ C, A ↔ T, C ↔ G, or G ↔ T) accounted for 51.9% (Table S4). Transitions between C ↔ T and A ↔ G appeared to be most prevalent, with each representing approximately 24.3% and 23.8% of the total polymorphisms. In contrast, C ↔ G transversions were the least common type of change, representing only 5.7% of the total polymorphisms. We categorized the SNPs according to their location on the reference genome. More than half of the GBS-based SNPs were distributed in intergenic regions (50.2%), whereas 35.2% of SNPs were distributed in introns, and 14.6% of SNPs were distributed in exons.

Segregation analysis of the polymorphic RAD loci showed that 8743 segregated in the progeny, and these were used in the subsequent linkage analysis. Of these genotyped RAD loci, 4041 were present in the lm × ll configuration, 3635 were in the nn × np configuration, 631 were in the ef × eg configuration (three alleles, both parents are heterozygous), 281 were in the ab × cd configuration (four alleles, both parents are heterozygous), and 155 were in the hk × hk configuration (two alleles, both parents are heterozygous). High missing values were observed among the genotyped RAD loci: the missing ratio ranged from 0.9% to 99.1%, with an average of 61.6%.

### Genetic linkage map

**Figure 1:**
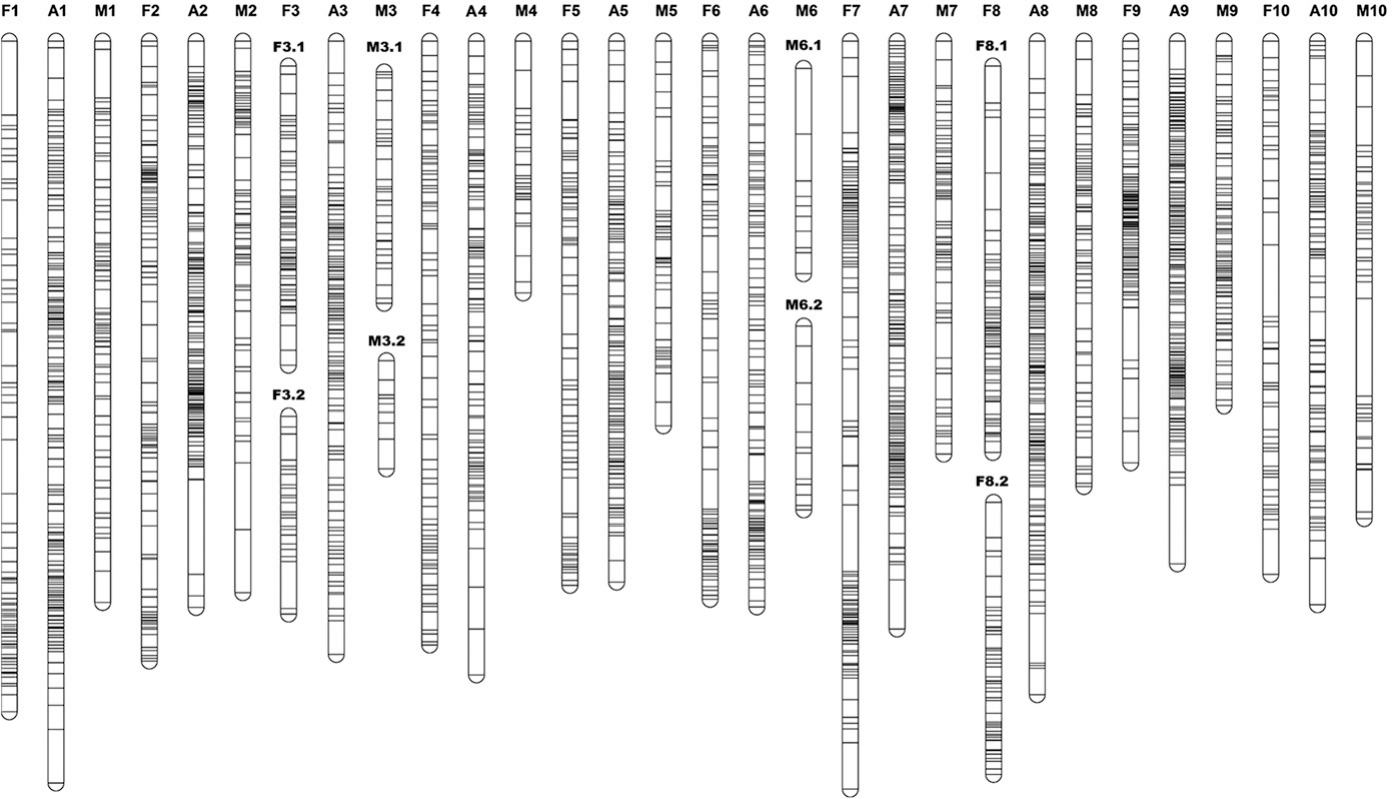
The sex-average and the sex-specific linkage maps, constructed using a hybridized family of *Crassostrea gigas* and *C. angulata*. The sex-average linkage groups are named A1–A10. The female linkage groups are named F1–F10. The male linkage groups are named M1–M10. The two linkage groups on the female map corresponding to A3 on the sex-average map are denoted F3.1 and F3.2, and represented as F3. The two linkage groups on the male map corresponding to A3 on the sex-average map are denoted M3.1 and M3.2, and represented as M3. M6.1 and M6.2 represent M6. F8.1 and F8.2 represent F8.

Following cosegregation analysis, 1694 RAD loci were mapped on the linkage map (mapped markers) (Figure 1, File S3, Table 1, Table S5, Table S6, and Table S7); the other 7049 RAD loci could not be mapped. A total of 3367 SNPs was located on the mapped markers, with an average of 1.9 SNPs per marker. Of the mapped markers, 930 were in the lm × ll configuration, 553 were in the nn × np configuration, 150 were in the ef × eg configuration, and 61 were in the ab × cd configuration.

**Table 1:** Length, number of markers, and average distance between markers for sex-average and sex-specific maps, as well as the female:male ratio, by linkage group, constructed using a hybridized family of *Crassostrea gigas* and *C. angulata*

	Number of Markers			Size (cM)				Average Distance Between Markers (cM)		
Linkage Group	Female Map	Male Map	Sex-Average Map	Female Map	Male Map	Sex-Average Map	F:M Ratio	Female Map	Male Map	Sex-Average Map
1	83	80	178	121.4	101.6	134.3	1.19	1.6	1.4	0.8
2	131	82	189	112.2	88.4	102.5	1.27	1.1	1.3	0.6
3	106	53	152	89.9	61.4	111	1.46	1.2	1.9	0.8
4	93	33	118	109.3	45.6	114.7	2.4	1.4	1.8	1.1
5	95	62	147	98.5	69.6	97.9	1.42	1.3	1.6	0.8
6	109	41	143	101.	70.5	102.4	1.43	1.2	3.6	0.9
7	144	86	215	116	74.7	106.4	1.55	1.	1.1	0.6
8	130	91	206	119.2	80.6	118.4	1.48	1.1	1.2	0.7
9	139	108	225	70.6	66.	94.7	1.07	0.6	0.7	0.5
10	72	53	121	96.5	86.4	102	1.12	1.9	1.9	1
Total	1102	689	1694	1034.6	744.8	1084.3	1.39	1.3	1.7	0.8

Initial sex-specific maps were constructed using the original markers, which contained missing data. The average number of expected recombination events per linkage group was 6.7 for the female linkage map, and 3.4 for the male linkage map (Table S8), indicating double recombination events due to missing values and erroneous marker genotypes. An imputation was performed using Maskov, to impute missing values and remove suspected erroneous genotypes. Following the imputation, the average number of expected recombination events per linkage group was 2.6 for the female linkage map, and 1.7 for the male linkage map, a reduction in recombination of 61.2% and 50%, respectively. For the genetic distance of each linkage group, a mean of 65% reduction for the female map, and a mean of 52.7% reduction for the male map was observed after the imputation, respectively. The imputed markers in the lm × ll and nn × np configurations, together with the original mapped markers in the ab × cd and ef × ef, configurations, were used to construct the sex-average map.

For the sex-average map, 1694 markers fell into 10 linkage groups (the linkage groups were named A1–A10) (Figure 1 and Table S9). For the female linkage map, 1102 markers fell into 12 linkage groups (the linkage groups were named F1–F10) (Figure 1 and Table S9). For the male linkage map, 689 markers fell into 12 linkage groups (the linkage groups were named M1–M10) (Figure 1 and Table S9). Analysis of common markers between the sex-specific and sex average maps revealed the correspondence of their linkage groups (Figure 1 and Table S9). Each of LG A1, A2, A4, A5, A7, A9, and A10 on the sex-average map corresponded to a single linkage group on the sex-specific map. LG F3.1, F3.2, and LG M3.1, M3.2 corresponded to LG A3 on the sex-average map. LG M6.1, M6.2 corresponded to LG A6 on the sex-average map. LG F8.1, F8.2 corresponded to LG A8 on the sex-average map. For the sex-specific map, linkage groups corresponding to the same sex-average group were considered the same linkage group, and the sum of the length of the linkage groups represented the total length of the corresponding linkage group. For example, for the female map, the length of LG F3 was the sum of LG F3.1 and LG F3.2 (Table 1). The sex-average map spanned a total genetic distance of 1084.3 cM, with an average spacing of 0.8 cM and a Coa of 98.7%; the length of each LG ranged from 94.7 cM (LG A9) to 134.3 cM (LG A1), with an average genetic length of 108.4 cM. The female map spanned a total genetic distance of 1034.6 cM, with an average spacing of 1.3 cM and a Coa of 97.5%; the length of each LG ranged from 70.6 cM (LG F9) to 121.4 cM (LG F1), with an average genetic length of 103.5 cM. The male map spanned a total genetic distance of 744.8 cM, with an average spacing of 1.7 cM and a Coa of 95.2%; the length of each LG ranged from 45.6 cM (LG M4) to 101.6 cM (LG M1), with an average genetic length of 74.5 cM.

We observed an overall female-to-male recombination ratio of 1.39:1 (Table 1). The largest recombination ratio between sexes was found for linkage group 4, with a female:male ratio of 2.40:1, and the smallest recombination ratio between sexes was found for linkage group 9, with a female:male ratio of 1.07:1. Localized differences in recombination rates between the sexes were observed. High recombination rates were found near the centromeric region on LG F1, F2, F4, F5, F6, F7, F8.1, F8.2, and F10 on the female map, and high recombination rates were found near the telomeric regions on LG M1, M2, M4, M5, M7, M8, M9, and M10 for the male map (Figure 1).

Of the 1694 markers on the sex-average map, 836 (49.4%) showed significant (*P* < 0.05) segregation distortion (Table S10 and File S4). For the sex-average linkage map, LG A8 and LG A5 presented the most severe segregation distortion, with 77.2% and 73.5% of markers distorted, respectively. LG A10 presented the lowest segregation distortion, with 12.4% of markers distorted. Distorted markers tended to be clustered, rather than randomly distributed across a linkage group (File S4). For the distorted markers in the lm × ll type (423), 183 showed homozygote deficiency while 240 showed heterozygote deficiency. For the distorted markers in the nn × np type (238), 120 showed homozygote deficiency, while 118 showed heterozygote deficiency.

### QTL mapping analysis of growth-related traits

A total of 27 QTL was identified, including seven for shell height, three for shell length, 10 for shell width, one for mass weight, and six for soft tissue weight (Table 2). These 27 QTL were distributed across eight linkage groups on the sex-average map, except for LG A3 and LG A6. More than half (16) of the QTL were clustered in their respective LGs. A major cluster containing five QTL (qGRT-8, qGRT-9, qGRT-18, qGRT-19, and qGRT-20) was detected in the narrow region between 67.3 and 76 cM on LG A8. On LG A2, another cluster containing four QTL (qGRT-13, qGRT-21, qGRT-22, and qGRT-23) was detected within a small region (59.3–66.5 cM). Three other clusters were situated between 44.4 and 47.4 cM of LG A1, which contained two QTL (qGRT-11 and qGRT-12), between 4.7 and 11.6 cM of LG A4, which contained three QTL (qGRT-1, qGRT-2, and qGRT-14), and between 44.3 and 53.9 cM of LG A7, which contained two QTL (qGRT-4 and qGRT-5). LG A8 contained the most QTL, with six, and LG A10 contained the fewest QTL, with one. Of the five growth-related traits, four had QTL in LG A8, which indicated that LG A8 shows enrichment for genes regulating growth. In particular, qGRT-7 for shell height and qGRT-6 and qGRT-25 for soft tissue weight were at the same location on LG A8. The phenotypic variance explained by these QTL ranged from 4.2% to 7.7%, with an average of 5.4%. The low phenotypic variance explained by these QTL indicated that no major loci (explaining >20% of the total variation) were detected. For shell height, the seven QTL explained 35.3% of the total phenotypic variance. For shell length, the three QTL explained 20.1% of the total phenotypic variance. For shell width, the 10 QTL explained 50.8% of the total phenotypic variance. For mass weight, the QTL explained 7.7% of the total phenotypic variance. For soft tissue weight, the six QTL explained 34.1% of the phenotypic variance. The LOD scores of the QTL varied from 3.4 to 5.1, with an average of 4.1. A number of genes related to growth-related traits were identified from the higher-density map based on the Pacific oyster genome assembly. As shown in Table S11, 38 annotated genes were identified in the QTL regions. Among the annotated genes, two were found to be growth-related (*agl* and *fbp1*), which function as key factors in carbohydrate metabolism, and others were found to participate in the assembly and regulation of the actin cytoskeleton (*avil*, *fmn2*, and *specc1l*), signal transduction pathways (*prkg1*, *dusp6*, and *grk1*), and the regulation of cell differentiation and development (*tbata* and *megf8*).

**Table 2:** Characteristics of quantitative trait loci for growth-related traits

Trait	QTL	Linkage Group	Map Distance (cM)	LOD	Variance Explained (%)
Shell height	qGRT-1	A4	4.7–5.9	3.6	4.4
	qGRT-2	A4	11.6	3.8	4.5
	qGRT-3	A7	38.1	3.4	4.5
	qGRT-4	A7	44.3–49.9	5.1	6.5
	qGRT-5	A7	51.9–53.9	4.8	6.1
	qGRT-6	A8	19.1–20	4	4.7
	qGRT-7	A10	79.4–79.9	3.9	4.6
Shell length	qGRT-8	A8	67.3–68.9	4.9	7.3
	qGRT-9	A8	71.7–73.9	4.6	6.7
	qGRT-10	A9	24.7	4.1	6.1
Shell width	qGRT-11	A1	44.4	3.8	4.6
	qGRT-12	A1	47.4	4.1	5
	qGRT-13	A2	65.9–66.5	3.9	4.2
	qGRT-14	A4	7.9	3.5	4.4
	qGRT-15	A4	28.7–30.2	3.5	4.4
	qGRT-16	A5	70.7	3.7	4.2
	qGRT-17	A8	51.3–52	4.6	6.8
	qGRT-18	A8	73.2	3.7	5.6
	qGRT-19	A8	74.6	3.8	5.8
	qGRT-20	A8	75.7–76	3.9	5.8
Soft tissue weight	qGRT-21	A2	59.3–60.7	4	5.9
	qGRT-22	A2	63.8–64	4.1	6
	qGRT-23	A2	65.6–65.9	5	7.2
	qGRT-24	A5	30.4	3.6	4.9
	qGRT-25	A8	19.8	3.7	5
	qGRT-26	A9	39.6–41.3	4.1	5.1
Mass weight	qGRT-27	A9	70.9	4.6	7.7

## Discussion

### Genotyping-by-sequencing in oysters

RRL sequencing methods coupled with next-generation sequencing technologies are widely employed to genotype SNPs in vertebrates and plants (Andrews *et al.* 2016), but only a limited number of studies utilize them to call SNPs in mollusks (Liu *et al.* 2013; Richards *et al.* 2013; Chu *et al.* 2014; Li and He 2014; Jiao *et al.* 2014). In this study, GBS was first used to screen for high-quality, abundant SNPs for construction of a genetic map for oysters. *Eco*RI and *Hin*fI were selected for the construction of RRLs; restriction digestion produced DNA fragments of 270–470 bp, which were sequenced for SNP screening. The mapping locations of the GBS reads and the distribution of the SNPs located on them were generally in accordance with the results of *in silico* simulations, indicating high consistency between simulated and actual digestions. This result indicated that *Eco*RI and *Hin*fI could generate evenly distributed markers, and constituted a good restriction enzyme combination for the GBS analysis of oyster. An enrichment of GBS reads in exons was observed, which may result from using two methylation-sensitive enzymes to construct the libraries (Pootakham *et al.* 2015). Deep sequencing of GBS libraries was performed to reduce the negative effect of nonuniform distribution of sequence reads, which mainly originated from inaccurate DNA quantification in the GBS analysis. A total of 15,783 polymorphic RAD loci was identified in the mapping population, for which 40,445 SNPs were detected. Deep sequencing of each locus, at 91.5-fold for the male, 63.4-fold for the female, and 13.1-fold to 143.2-fold for the progeny, guaranteed the accuracy of genotyping.

Despite the deep sequencing of the RRL libraries, a high proportion of missing values (low consistency of RAD loci among individuals) was observed among the genotyped markers, which was unexpected. Several factors may explain the high missing data ratio. The first factor may be the unstable fidelity of the restriction enzymes used in the library construction. The activity of the restriction enzymes can be affected by sequence context, enzyme concentration and buffer composition. Unsuitable conditions may lead to either ineffective cleavage at the cognate restriction site or relaxed specificity allowing cleavage of degenerate ‘star’ sites (Kamps-Hughes *et al.* 2013). The second reason may be differences in methylation patterns between the two parents and the progeny, which would lead to inconsistencies in enzyme cut-site locations among individuals, resulting in the generation of inconsistent tags (Xia *et al.* 2014). The third reason may be the genomic differences between *C. gigas* and *C. angulata*. The fourth reason may be the nonuniform distribution of sequence reads among different individuals resulted from inaccurate quantification of DNA for restriction digestion and differentiated PCR amplification efficiency. Although high missing value ratio was observed, nearly 1600 RAD loci showed missing value ratio smaller than 30%, which could be used to construct linkage map. The application of GBS in oysters could significantly improve the throughput of SNP genotyping and reduce the cost of genetic map construction and population genetic analysis.

### Linkage mapping

Of the 8743 genotyped RAD loci, 1694 were successfully mapped to the linkage map while 7049 failed to be mapped to the map. The low ratio of RAD loci that can be assigned into the linkage map is also observed in other linkage mapping studies (Li and He 2014; Ren *et al.* 2016). In this study, the main reason for the unsuccessful mapping of the 7049 RAD loci may be their high missing ratio (with an average of 61.6%). Of the mapped 1694 markers, an average missing ratio of 26.8% was observed, which was comparable to other GBS studies (Ward *et al.* 2013). It was reported that missing values and typing errors in the data would reduce the proportion of correctly ordered map (Hackett and Broadfoot 2003). To reduce the negative effects of missing values and typing errors on map order, we used Maskov to impute missing values and correct typing errors for genetic markers (Ward *et al.* 2013). The successful imputation reduced linkage group length, and decreased the recombinant count for each linkage group on the sex-specific maps, indicating that the quality of the sex-specific maps was improved. The sex-average map built with the imputed markers thus represents reliable marker order and map distance.

Compared to the previously published linkage maps of the Pacific oyster constructed with first-generation DNA markers (Li and Guo 2004; Hubert and Hedgecock 2004; Hubert *et al.* 2009; Sauvage *et al.* 2010; Plough and Hedgecock 2011; Guo *et al.* 2012; Zhong *et al.* 2014), the genetic map in this study contains more markers and has higher density. More recently, Hedgecock *et al.* (2015) constructed second-generation linkage maps with high density for the Pacific oyster, mainly based on 1085 coding SNPs generated by EST sequencing, as well as 66 microsatellite DNA markers, with an average marker spacing of 1.0 cM. This map represented a substantial improvement on previously reported first-generation maps. In the present study, we constructed a second-generation linkage map based on SNPs generated through GBS, which is more efficient in terms of cost and time than microarray based and PCR-base genotyping. The sex-average map consisted of 1695 tags, with an average interval of 0.8 cM.

The number of linkage groups (10) on the sex-average map corresponded well to the haploid chromosome number of oysters (10). Although both the female and male maps contained 12 linkage groups, they corresponded well with the 10 linkage groups on the sex-average map. The discrepancy between the number of linkage groups and the haploid chromosome number observed for the sex-specific maps was also observed in previously published oyster linkage maps with relatively low marker number (Hubert and Hedgecock 2004; Li and Guo 2004; Guo *et al.* 2012). The split between the linkage groups is unexpected in terms of the high marker number and the small average interval in this study. It appears that, for the sex-specific maps, some markers that could have integrated the two linkage groups that aligned with a single linkage group on the sex-average map were missing. Potential reasons for the missing markers are as follows: 1) the gap may correspond to a genomic region that is homozygous in the parents; 2) the gap may correspond to a genomic region with high recombination rates but low marker density (Li and Guo 2004).

Both overall and localized differences in recombination rates between the sexes were observed in the present study. In both vertebrates and invertebrates, female maps are usually longer than male maps (Hansson *et al.* 2005; Lien *et al.* 2011; Jones *et al.* 2013). The same phenomenon was also observed in *C. gigas*. Hubert and Hedgecock (2004) observed a female-to-male recombination ratio of 1.25:1, and Li and Guo (2004) observed a female-to-male recombination ratio of 1.36:1. In contrast, Hedgecock *et al.* (2015) observed no significant differences between sexes in the recombination rates, which suggested that sex-specific recombination pattern varied among different mapping families. In this study, the overall female-to-male recombination ratio was 1.39:1, which was in accordance with that in previous studies. In addition to the overall recombination rate difference between the sexes, localized differences in recombination rates were also observed: high recombination rates were found near the centromeric region on the female map, and high recombination rates were found near the telomeric region on the male map. In other aquatic animals, such as rainbow trout (Sakamoto *et al.* 2000), zebrafish (Singer *et al.* 2002), and *Pinctada maxima* (Jones *et al.* 2013), a similar pattern was also observed. The mechanism of overall and localized recombination rate differences remains unclear. However, several possible causes have been proposed: first, the germ cells developed in different environments; second, meiosis was initiated at different time points; third, pairing and synapses of the homologs at meiosis differed between oocytes and spermatocytes. Further studies are required to reveal the mechanism governing the differences in recombination rates between the sexes.

### Segregation distortion

Segregation distortion is a common phenomenon in many species, and the proportion of distorted markers varies across different species (Graner *et al.* 1991; Tan *et al.* 2001; Li *et al.* 2005; Lallias *et al.* 2007). In this study, we observed 49.4% distorted markers in the outbred cross, which is comparable with previous studies. For the Pacific oyster, segregation distortion is observed in both inbred and outbred crosses. The rate of distorted markers in inbred crosses ranges from 17% to 66% (Launey and Hedgecock 2001; Sauvage *et al.* 2010; Plough and Hedgecock 2011; Plough 2012; Hedgecock *et al.* 2015), and the rate in outbred crosses ranges from 18.8% to 68% (Guo *et al.* 2012; Hedgecock *et al.* 2015; Plough *et al.* 2016). Segregation distortion has been reported to be caused by zygotic viability selection, duplicated genes, transposable elements, and unusual meiotic segregation distortion (Knox and Ellis 2002; Gut and Lathrop 2004; Hubert *et al.* 2010). Launey and Hedgecock (2001) demonstrated experimentally that a high genetic load of deleterious recessive genes, which resulted in strong zygotic selection during the larvae stage, was a cause of segregation distortion in the Pacific oyster. Another factor leading to the high proportion of distorted markers observed in this study may be the hybridization between *C. gigas* and *C. angulata*. Hybrid incompatibility is a key biological factor causing uneven transmission of alternate alleles, which is frequently caused by disrupted genetic interactions among loci of parental lineages, resulting in the nonrandom elimination of particular allelic combinations (Orr and Turelli 2001; Zhou *et al.* 2015). The ratio of markers presenting heterozygotes inviability (54.2%) is slightly bigger than that of markers presenting homozygotes inviability (45.8%) in this mapping population, which is in accordance with previous finding that heterozygotes are inviable in outbred families (Plough *et al.* 2016).

Hedgecock *et al.* (2015) reported that the effect of distorted markers on map construction is not universal, and is likely to vary within and among linkage maps. Considering that distorted markers can be potentially helpful in the detection of QTL (Xu 2008), and that discarding them could potentially remove massive amounts of information and decrease genome coverage (Luo *et al.* 2005), we retained the distorted markers for map construction despite their potential negative effects on the map. The uneven distribution of the distorted markers may suggest that marker distortion was not caused by technical limitations or other typing errors. In this study, the distorted markers were used in constructing the linkage map, as they represented nearly half of all mapped markers and revealed genomic regions subject to viability selection.

### QTL for growth-related traits and associated genes

The high density of the genetic map allowed us to perform QTL fine mapping of economically important growth-related traits. In the present study, 27 QTL associated with growth-related traits of oysters were found to be distributed in eight LGs. Interestingly, more than half of the QTL were concentrated within narrow regions (clusters) in the LGs, which indicated clustering of genes regulating growth in oysters. In previous studies, a limited number of QTL for growth-related traits were detected based on the first-generation genetic maps with low density. For example, only three QTL were identified for two principal components, each of which explained 0.6%, 7.5%, and 13.8%, respectively, of the phenotypic variance based on a genetic map constructed using SSR and AFLP markers, and no linked candidate genes were identified (Guo *et al.* 2012). In the present study, the phenotypic variances explained by these QTL were relatively low (with an average of 5.4%), which indicated that no major loci were detected and the growth-related traits were regulated by many genes of small effect. The low phenotypic variances explained by detected QTL were also observed in the disease-resistance QTL mapping (5.1–8.38%) of the Japanese flounder (Shao *et al.* 2015). The identified QTL explain relatively low phenotypic variations, and more fine-mapping QTL based on multiple families are required before they can be utilized in the molecular breeding practice.

In the present study, within the QTL regions, 38 genes were identified, and two were previously reported to be associated with growth. The glycogen debrancher enzyme encoded by *agl* is important for degradation of glycogen, the major reserve for glucose storage; it is known to play a central role in providing energy for maintenance and gametogenic development (Li *et al.* 2006). *Agl* was also identified as being associated with glycogen content in the Pacific oyster (She *et al.* 2015). The fructose-1,6-bisphosphatase encoded by *fba* is a key enzyme in glucose metabolism, and is essential for cell growth (Brooks and Storey 1988; Mazurek *et al.* 1998; Singh *et al.* 2004; Campanella *et al.* 2005). It catalyzes the reversible cleavage of fructose-1,6-bisphosphate to glyceraldehyde-3-phosphate and dihydroxyacetone phosphate (Lu *et al.* 2004). One SNP in *fba* has been identified to present a significant correlation with growth traits in *Meretrix meretrix* (Wang *et al.* 2012a). In addition to *agl* and *fba*, several genes participating in the assembly and regulation of the actin cytoskeleton (*avil*, *fmn2*, and *specc1l*) were also identified within the QTL regions. A genome-wide association study of calf birth weight in Holstein cattle using SNPs revealed the enrichment of the “Regulation of actin cytoskeleton” pathway, as defined by the Kyoto Encyclopedia of Genes and Genomes, indicating an association between growth-related traits and actin cytoskeleton regulation in bovines (Cole *et al.* 2014). The QTL mapping results reported here also revealed a correlation between growth-related traits and actin cytoskeleton regulation in oysters. Other genes identified in QTL regions included *prkg1*, *dusp6*, and *grk1*, which participate in signal transduction, and *tbata* and *megf8*, which participate in regulation of cell differentiation and development. These genes are valuable candidate growth-related genes, and warrant further investigation to confirm their involvement.

### Conclusion

In this study, abundant SNPs were successfully obtained via GBS. A high-density linkage map was constructed for a hybrid mapping family of *C. gigas* × *C. angulata*. A total of 27 QTL was detected for five growth-related traits and 38 associated genes were identified, which will provide valuable genetic resources and the basis for MAS for both *C. gigas* and *C. angulata*.

## Supplementary Material

Supplemental Material
