# Supplementary material for: A High-Density SNP Genetic Linkage Map and QTL Analysis of Growth-Related Traits in a Hybrid Family of Oysters (Crassostrea gigas × Crassostrea angulata) Using Genotyping-by-Sequencing
Source: G3 (Bethesda). 2016 Mar 17;6(5):1417–26. doi: 10.1534/g3.116.026971 (PMC4856092; doi:10.1534/g3.116.026971)
Supplement: Supplemental Material [file supp_6_5_1417__index.html]

A High-Density SNP Genetic Linkage Map and QTL Analysis of Growth-Related Traits in a Hybrid Family of Oysters (Crassostrea gigas × Crassostrea angulata) Using Genotyping-by-Sequencing — Supplemental Material 

# A High-Density SNP Genetic Linkage Map and QTL Analysis of Growth-Related Traits in a Hybrid Family of Oysters (*Crassostrea gigas × Crassostrea angulata*) Using Genotyping-by-Sequencing

## Supplemental Material for Wang, Li, and Zhang, 2016

**Files in this Data Supplement:**

- File S1 - Histograms of five growth-related traits of a hybridized family of *Crassostrea gigas* and *C. angulate*. (.pdf, 25 KB)
- File S2 - Boxplots of comparisons of the five growth-related traits between female and male progeny of a hybridized family of *Crassostrea gigasM* and *C. angulate*. (.pdf, 21 KB)
- File S3 - The 10 linkage groups of the sex-average map, constructed using a hybridized family of *Crassostrea gigas* and *C. angulate*. (.pdf, 4906 KB)
- File S4 - The distribution of distorted markers in each linkage group on the sex-average map, constructed using a hybridized family of *Crassostrea gigas* and *C. angulate*. (.pdf, 20 KB)
- Table S1 - Five growth-related traits and gender for every progeny of the hybridized family of *Crassostrea gigas* and *C. angulate*. (.xlsx, 15 KB)
- Table S2 - Correlations between the five growth-related traits in a hybridized family of *Crassostrea gigas* and *C. angulate*. (.xlsx, 9 KB)
- Table S3 - Details of the sequencing and genotyping results of the six genotyping-by-sequencing libraries for the hybridized family of *Crassostrea gigas* and *C. angulate*. (.xlsx, 18 KB)
- Table S4 - Summary of single nucleotide substitutions identified in a hybridized family of *Crassostrea gigas* and *C. angulate*. (.xlsx, 10 KB)
- Table S5 - Genotypes of 1695 mapped markers in 106 progeny of a hybridized family of *Crassostrea gigas* and *C. angulate*. (.xlsx, 644 KB)
- Table S6 - Sequences of 1695 mapped SNP tags produced by genotyping-by-sequencing in a hybridized family of *Crassostrea gigas* and *C. angulate*. (.xlsx, 91 KB)
- Table S7 - Markers and their positions on the 10 linkage groups of the sex-average map constructed using a hybridized family of *Crassostrea gigas* and *C. angulate*. (.xlsx, 55 KB)
- Table S8 - The comparison of genetic distances and expected recombination counts before and after the imputation for the sex-specific maps, by linkage group. (.xlsx, 10 KB)
- Table S9 - Length and number of markers for the sex-specific maps, by linkage group, and their correspondence to the linkage groups of the sex-average map. (.xlsx, 10 KB)
- Table S10 - Proportion of distorted markers for each linkage group of the sex-average map of a hybridized family of *Crassostrea gigas* and *C. angulate*. (.xlsx, 10 KB)
- Table S11 - Annotated growth-related genes determined by QTL analysis of oysters. (.xlsx, 11 KB)
