# Supplementary material for: A High-Density SNP Genetic Linkage Map and QTL Analysis of Growth-Related Traits in a Hybrid Family of Oysters (Crassostrea gigas × Crassostrea angulata) Using Genotyping-by-Sequencing
Source: G3 (Bethesda). 2016 Mar 17;6(5):1417–26. doi: 10.1534/g3.116.026971 (PMC4856092; doi:10.1534/g3.116.026971)
Supplement: Supplemental Material [file supp_g3.116.026971_FileS1.pdf]

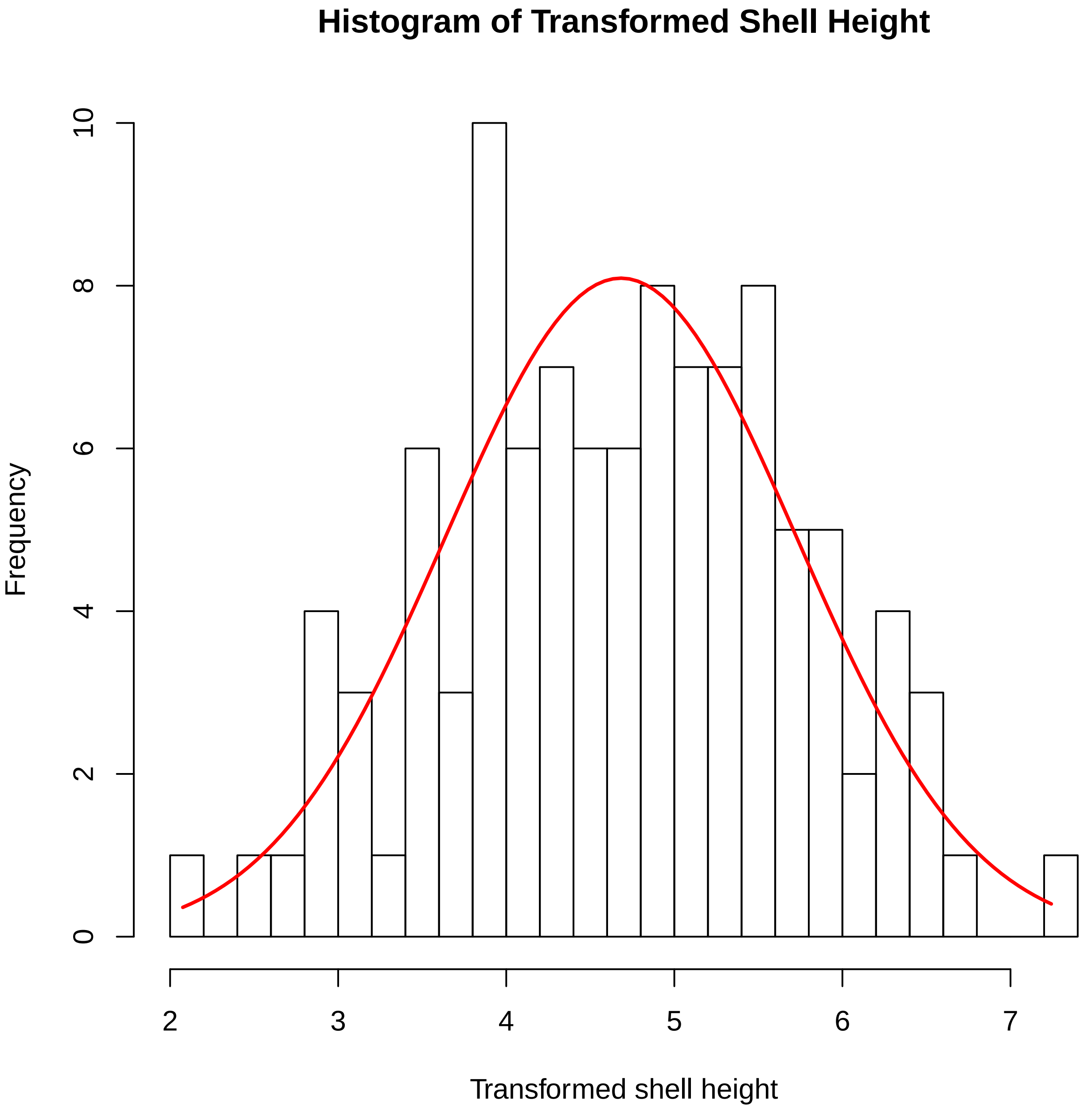

Histogram of Transformed Shell Height
Frequency
0
2
4
6
8
10
2
3
4
5
6
7
Transformed shell height

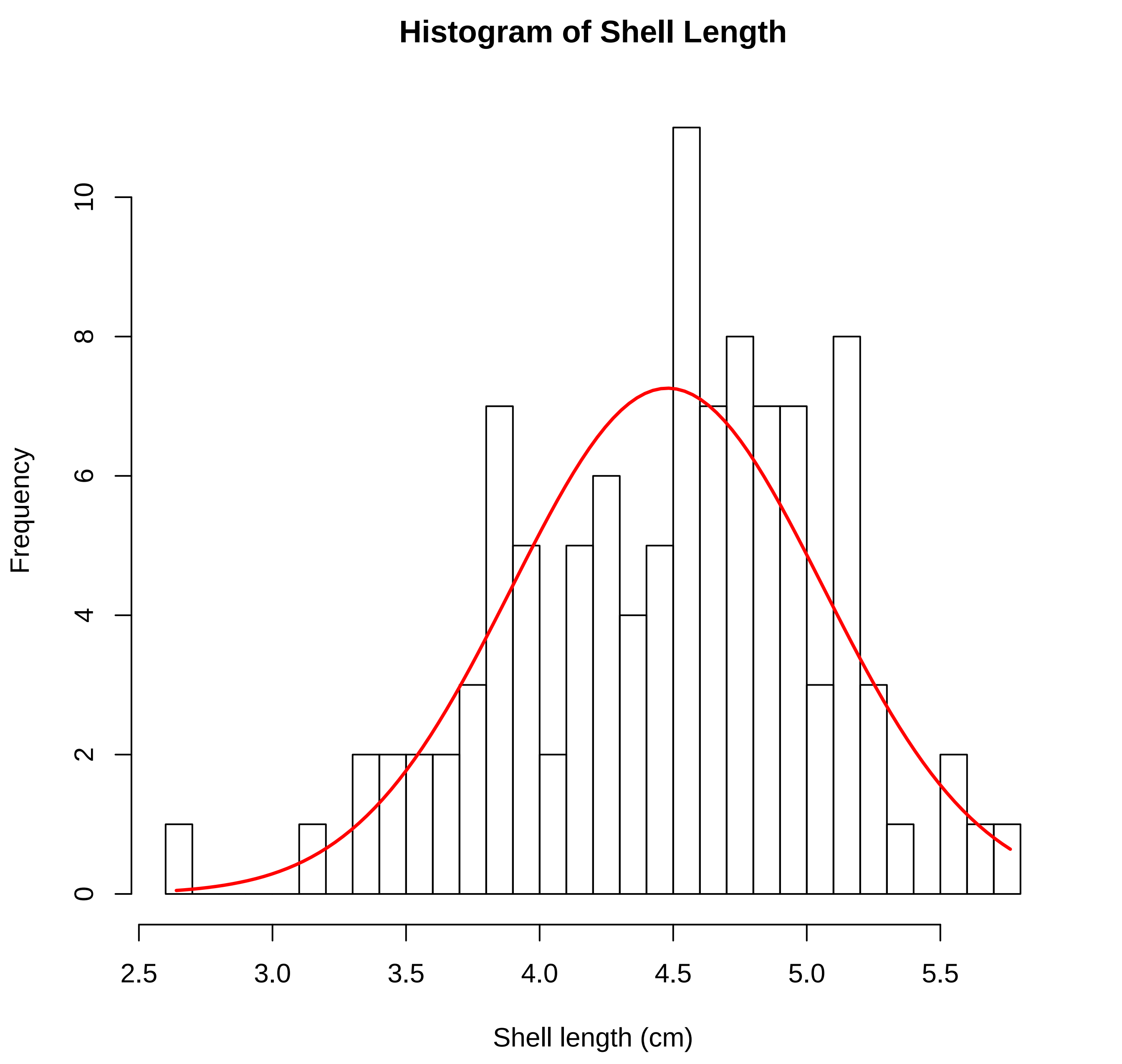
Histogram of Shell Length
Frequency
0
2
4
6
8
10
2.5
3.0
3.5
4.0
4.5
5.0
5.5
Shell length (cm)

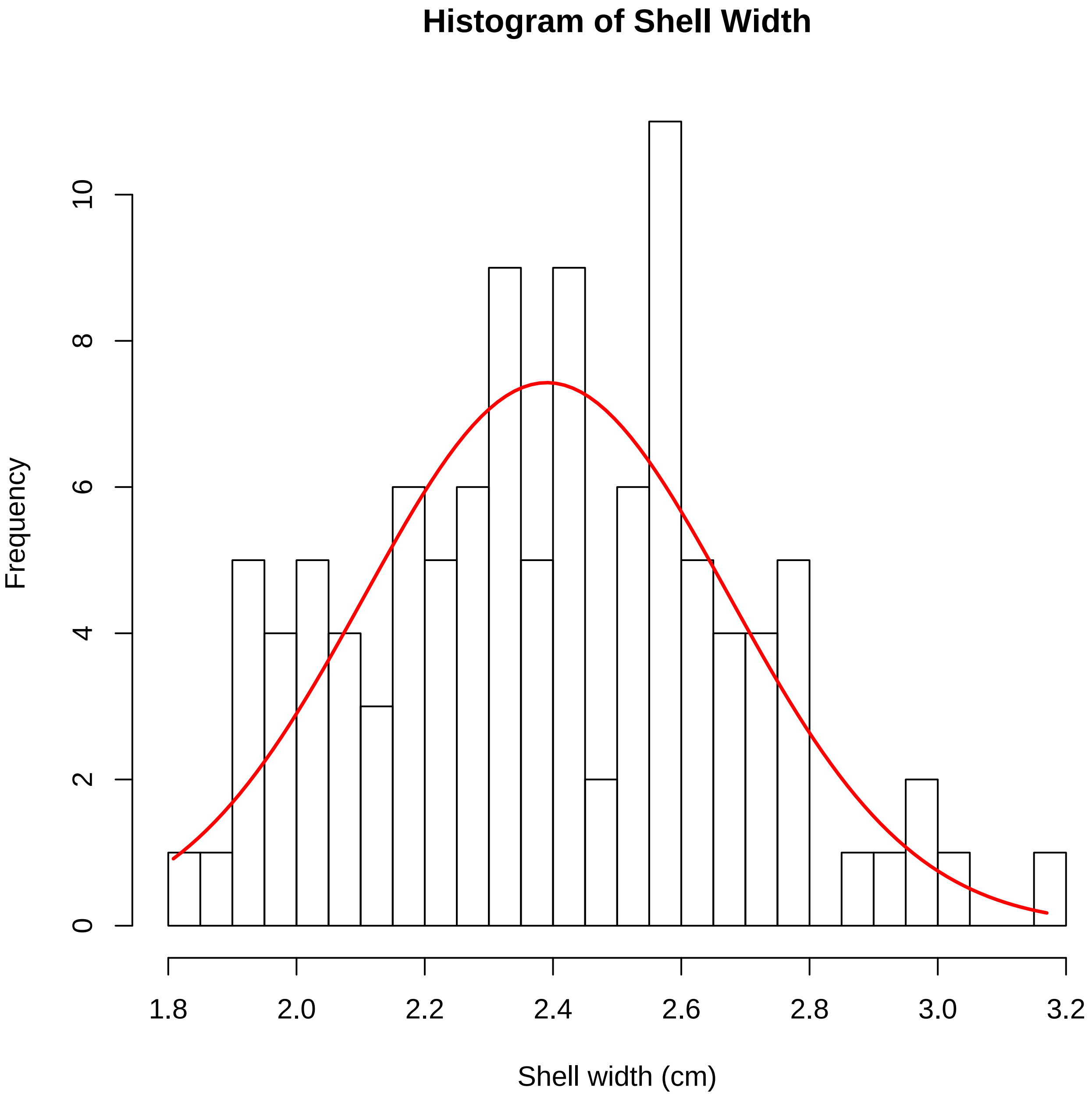
Histogram of Shell Width
Frequency
0
2
4
6
8
10
1.8
2.0
2.2
2.4
2.6
2.8
3.0
3.2
Shell width (cm)

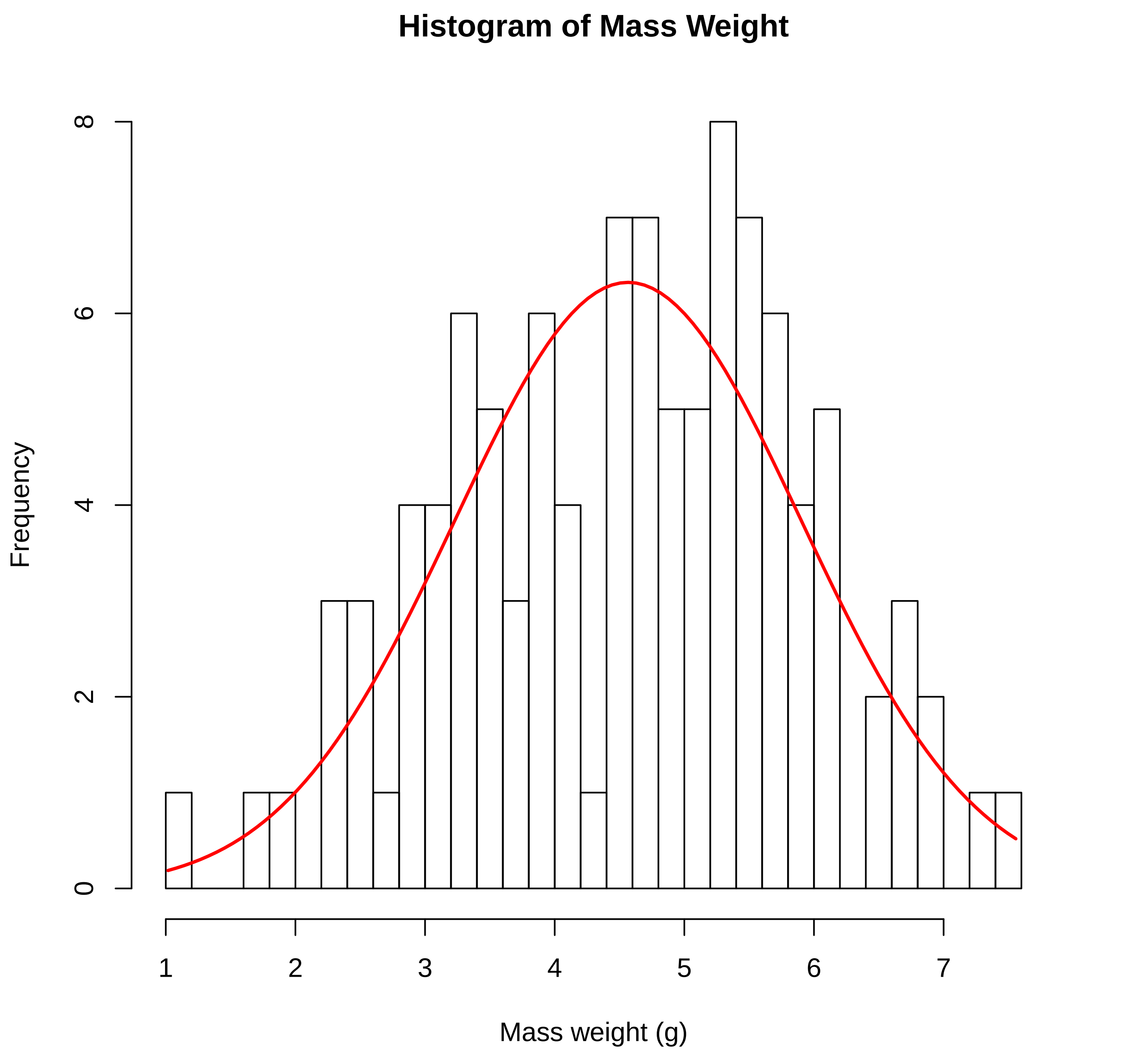

Histogram of Mass Weight
Frequency
0
2
4
6
8
1
2
3
4
5
6
7
Mass weight (g)

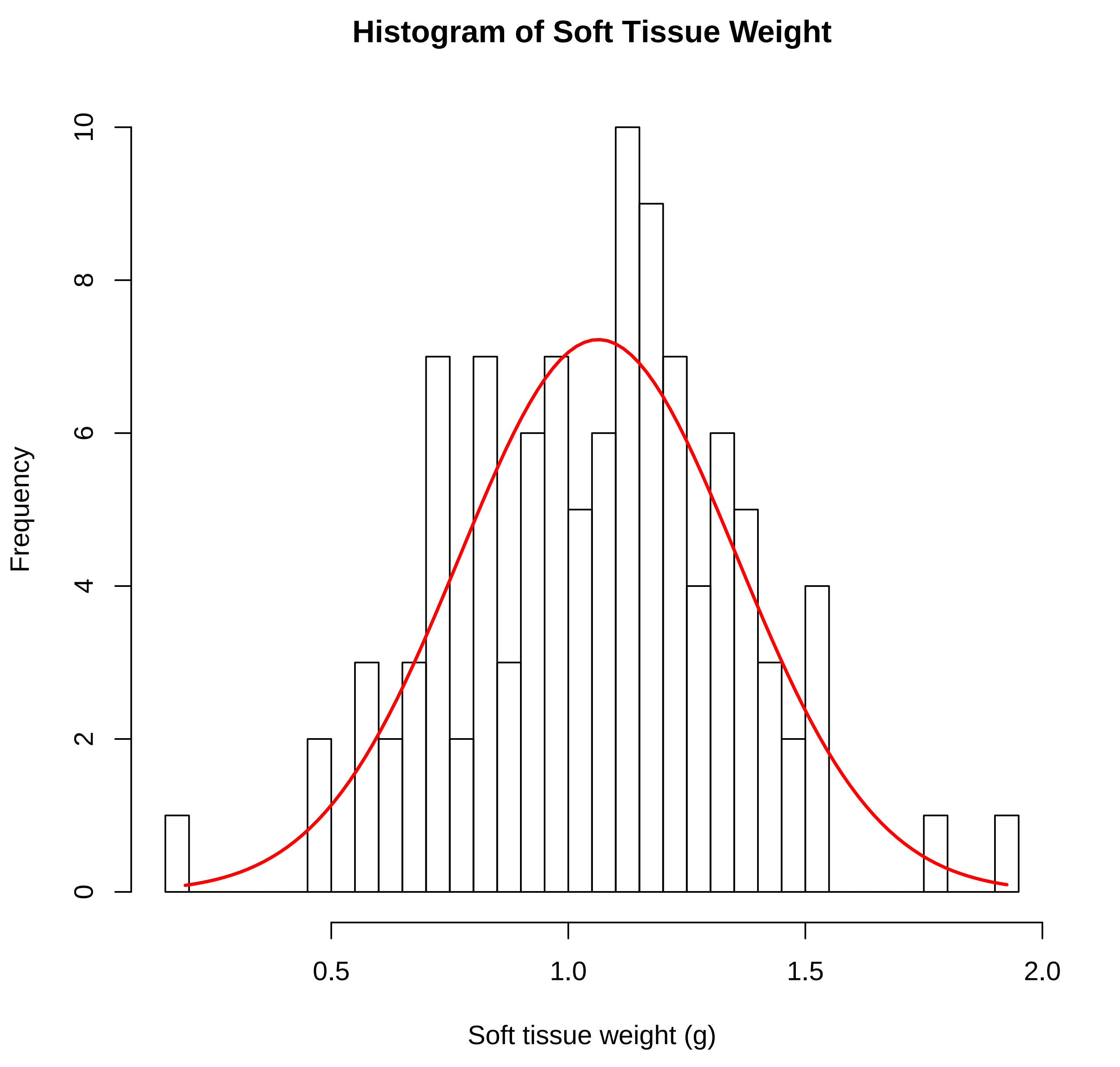

Histogram of Soft Tissue Weight
Frequency
0
2
4
6
8
10
0.5
1.0
1.5
2.0
Soft tissue weight (g)
