# Supplementary material for: A High-Density SNP Genetic Linkage Map and QTL Analysis of Growth-Related Traits in a Hybrid Family of Oysters (Crassostrea gigas × Crassostrea angulata) Using Genotyping-by-Sequencing
Source: G3 (Bethesda). 2016 Mar 17;6(5):1417–26. doi: 10.1534/g3.116.026971 (PMC4856092; doi:10.1534/g3.116.026971)
Supplement: Supplemental Material [file supp_g3.116.026971_FileS2.pdf]

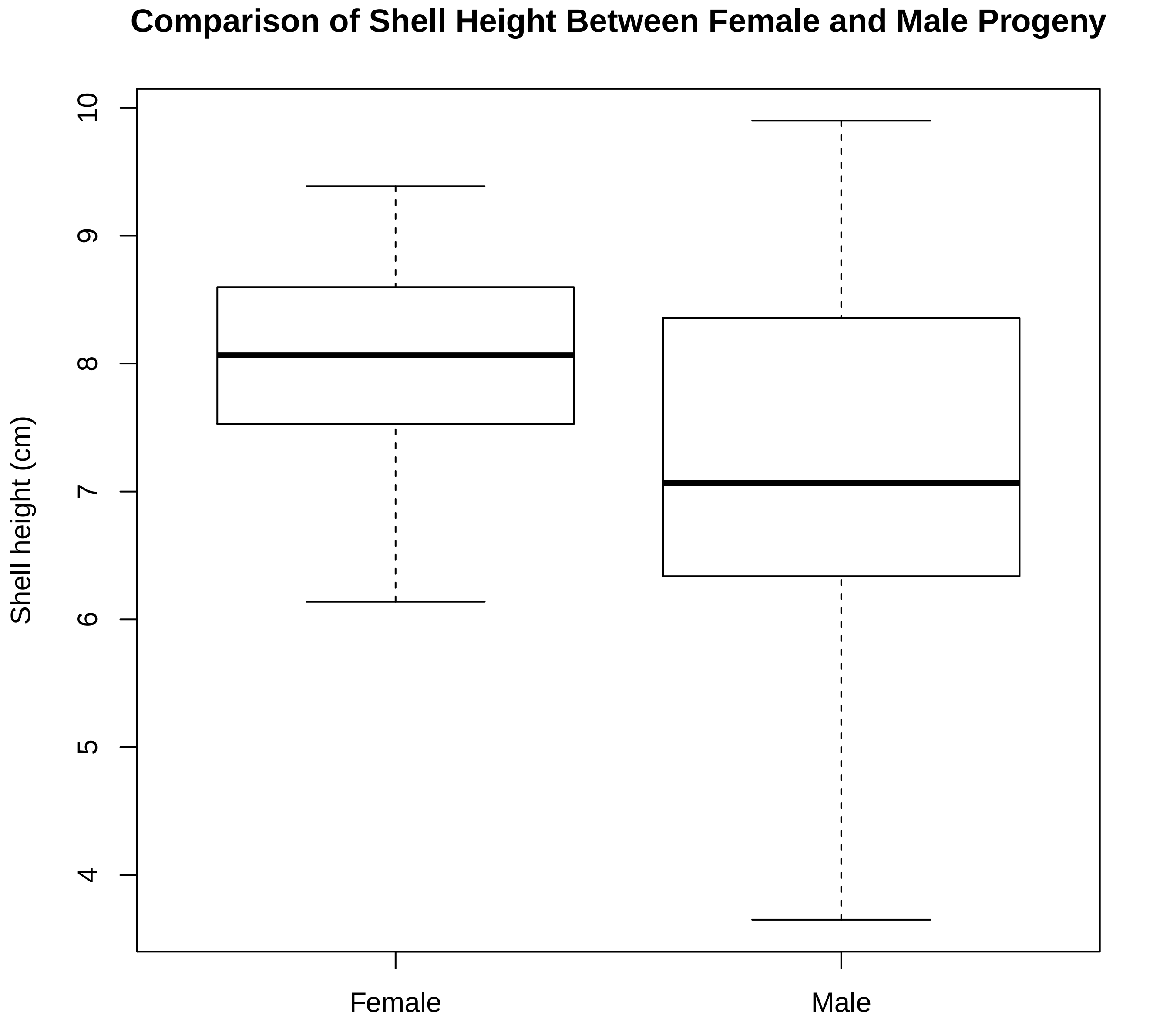

Comparison of Shell Height Between Female and Male Progeny
Shell height (cm)
10
9
8
7
6
5
4
Female
Male

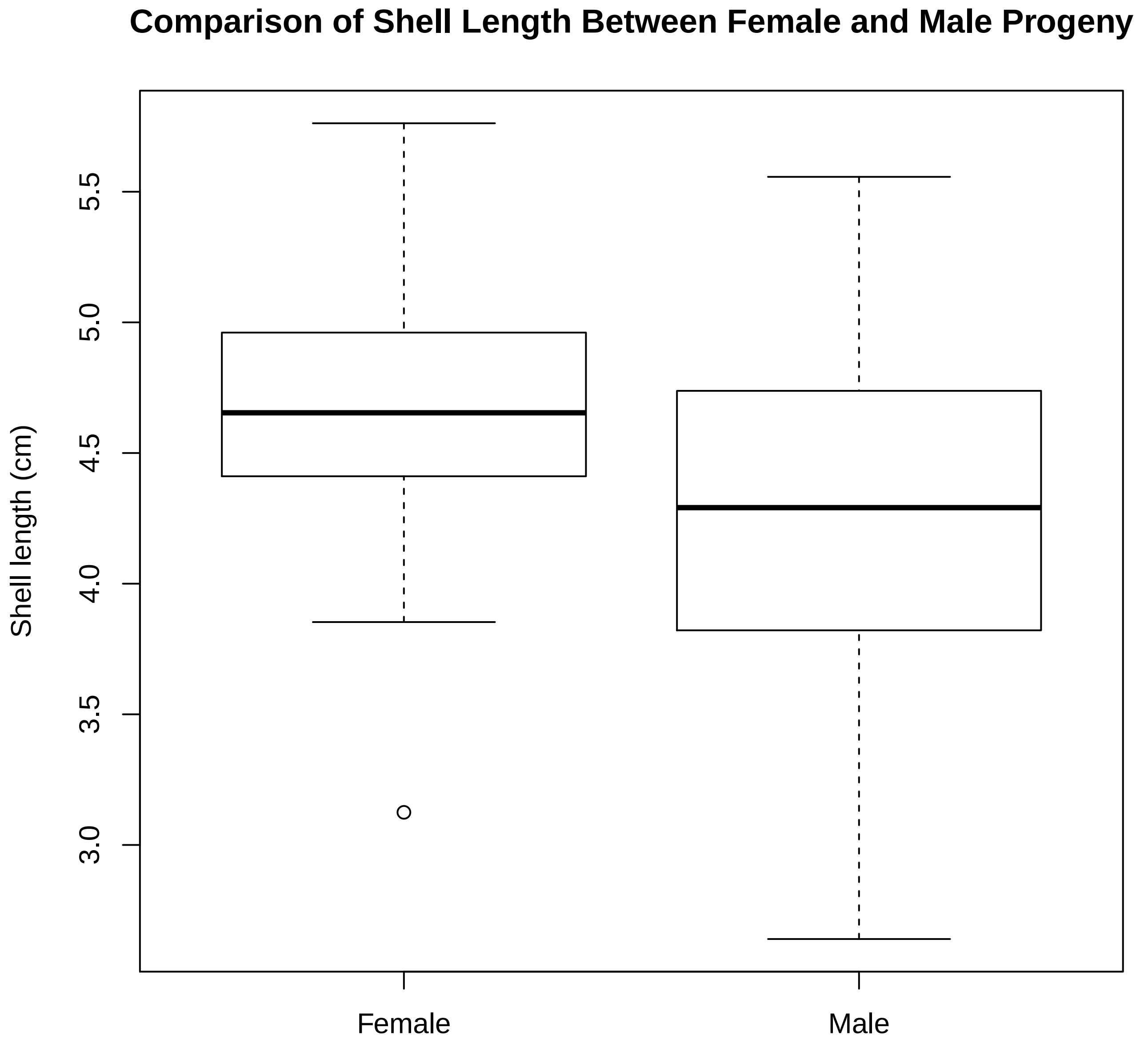

Comparison of Shell Length Between Female and Male Progeny
Shell length (cm)
3.0
3.5
4.0
4.5
5.0
5.5
Female
Male

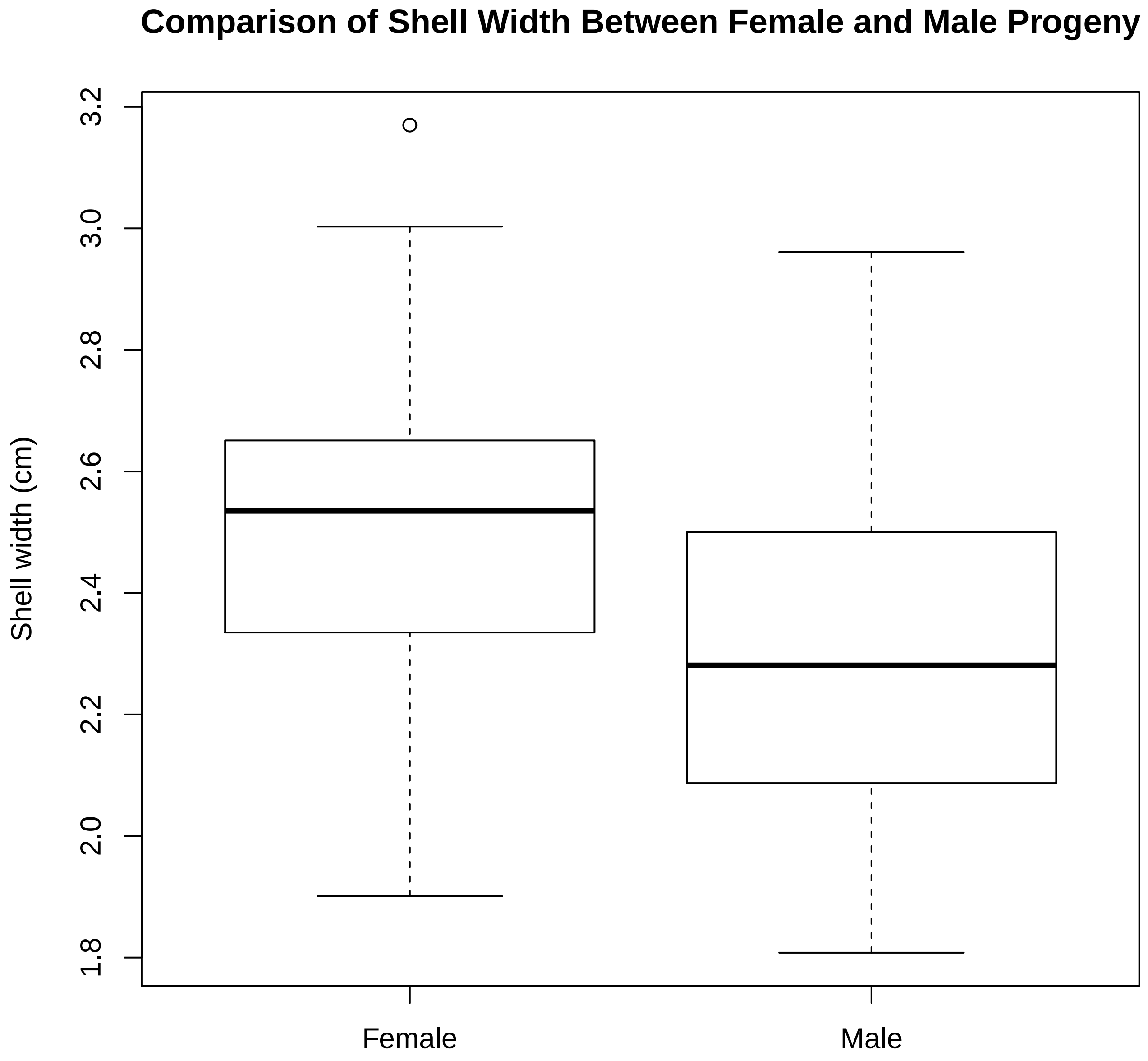

Comparison of Shell Width Between Female and Male Progeny
Shell width (cm)
3.2
3.0
2.8
2.6
2.4
2.2
2.0
1.8
Female
Male

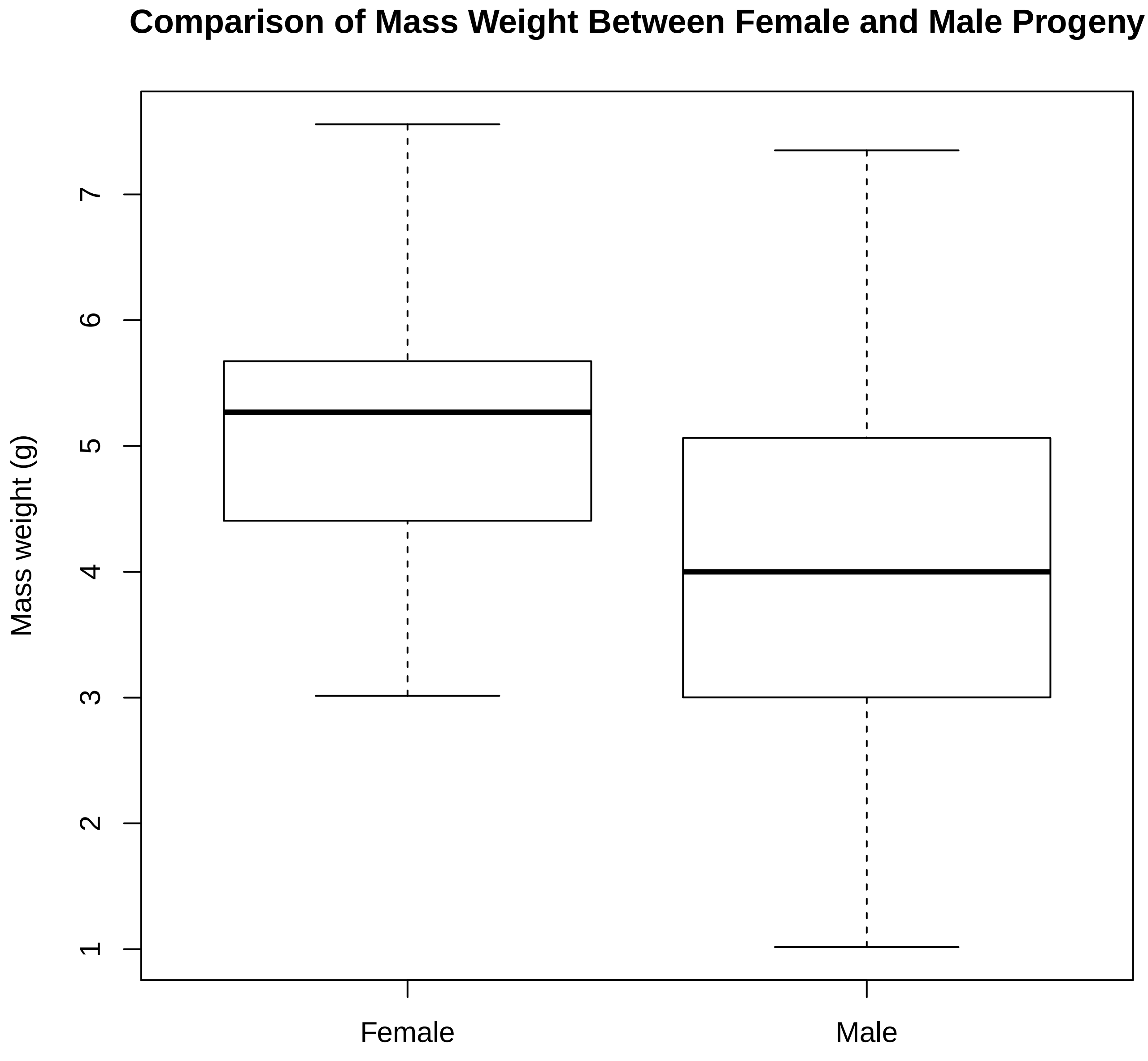
Comparison of Mass Weight Between Female and Male Progeny
Mass weight (g)
1
2
3
4
5
6
7
Female
Male

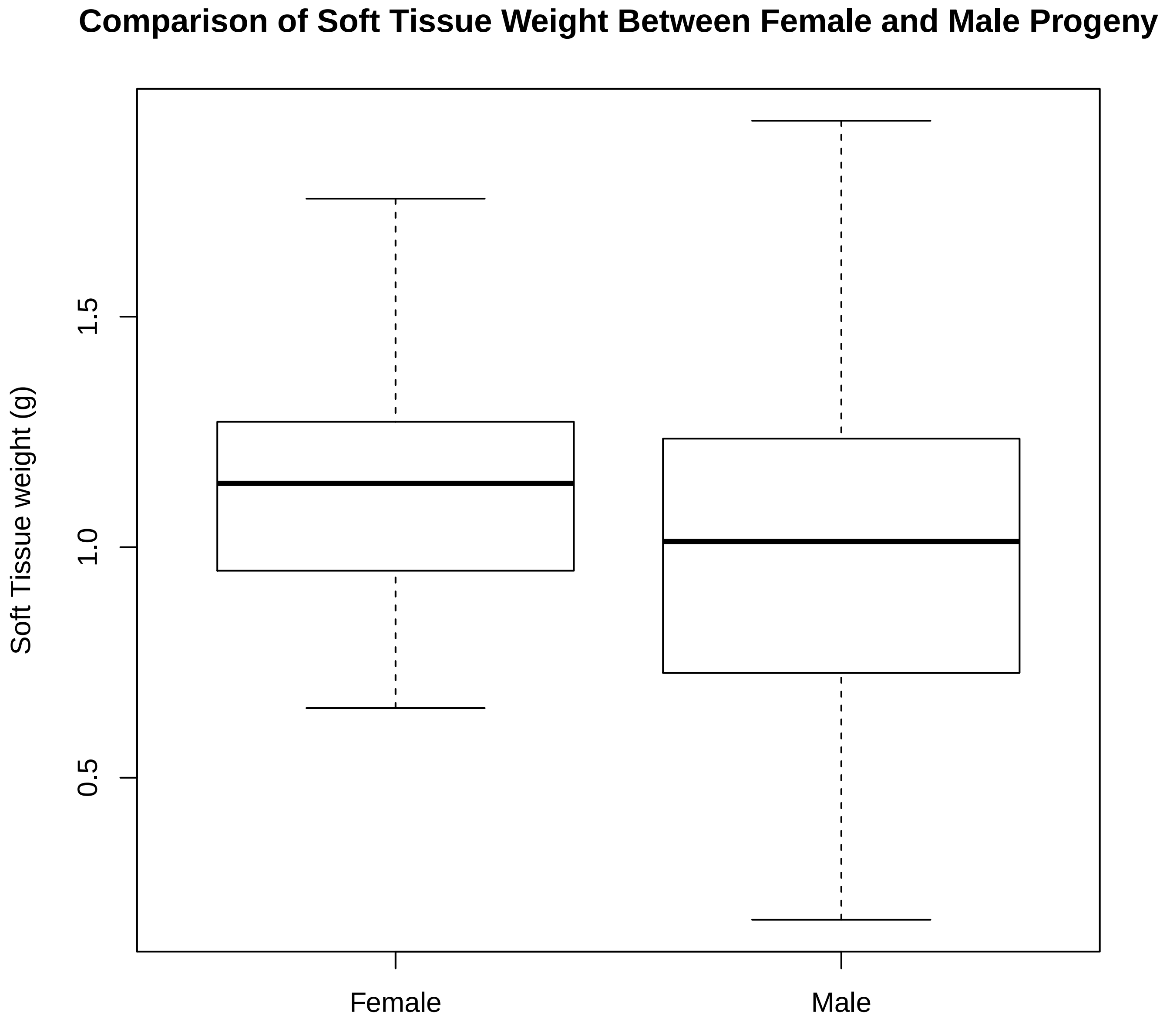
Comparison of Soft Tissue Weight Between Female and Male Progeny
Soft Tissue weight (g)
0.5
1.0
1.5
Female
Male
