# Supplementary material for: A High-Density SNP Genetic Linkage Map and QTL Analysis of Growth-Related Traits in a Hybrid Family of Oysters (Crassostrea gigas × Crassostrea angulata) Using Genotyping-by-Sequencing
Source: G3 (Bethesda). 2016 Mar 17;6(5):1417–26. doi: 10.1534/g3.116.026971 (PMC4856092; doi:10.1534/g3.116.026971)
Supplement: Supplemental Material [file supp_g3.116.026971_FileS3.pdf]

LG A1

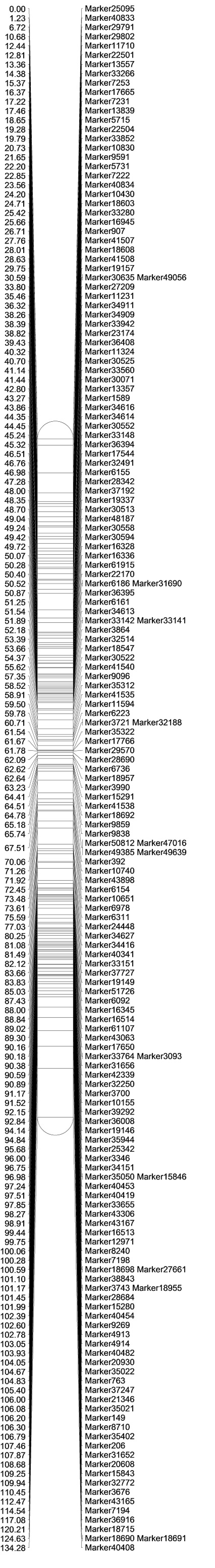
0.00
1.23
6.72
10.68
12.44
12.81
13.36
14.38
15.37
16.37
17.22
17.46
18.65
19.28
19.79
20.73
21.65
22.20
22.85
23.56
24.20
24.71
25.42
25.66
26.71
27.76
28.01
28.63
29.75
30.59
33.80
35.46
36.32
38.26
38.39
38.82
39.43
40.32
40.70
41.14
41.44
42.80
43.27
43.86
44.35
44.45
45.24
45.32
46.51
46.76
46.98
47.28
48.00
48.35
48.70
49.04
49.24
49.42
49.72
50.07
50.28
50.40
50.52
50.87
51.25
51.54
51.89
52.18
53.39
53.66
54.37
55.62
57.35
58.52
58.91
59.50
59.78
60.71
61.54
61.67
61.78
62.09
62.62
62.64
63.23
64.41
64.51
64.78
65.18
65.74
67.51
70.06
71.26
71.92
72.45
73.48
73.61
75.59
77.03
80.25
81.08
81.49
82.12
83.66
83.83
85.03
87.43
88.00
88.84
89.02
89.30
90.16
90.18
90.38
90.59
90.89
91.17
91.52
92.15
92.84
94.14
94.84
95.68
96.00
96.75
96.98
97.24
97.51
97.85
98.27
98.91
99.44
99.75
100.06
100.28
100.59
101.10
101.17
101.45
101.99
102.39
102.60
102.78
103.05
103.93
104.05
104.67
104.83
105.40
106.00
106.08
106.20
106.30
106.79
107.46
107.87
108.68
109.25
109.94
110.45
112.47
114.54
117.08
120.21
124.63
134.28
Marker25095
Marker40833
Marker29791
Marker29802
Marker11710
Marker22501
Marker13557
Marker33266
Marker7253
Marker17665
Marker7231
Marker13839
Marker5715
Marker22504
Marker33852
Marker10830
Marker9591
Marker5731
Marker7222
Marker40834
Marker10430
Marker18603
Marker33280
Marker16945
Marker907
Marker41507
Marker18608
Marker41508
Marker19157
Marker30635 Marker49056
Marker27209
Marker11231
Marker34911
Marker34909
Marker33942
Marker23174
Marker36408
Marker11324
Marker30525
Marker33560
Marker30071
Marker13357
Marker1589
Marker34616
Marker34614
Marker30552
Marker33148
Marker36394
Marker17544
Marker32491
Marker6155
Marker28342
Marker37192
Marker19337
Marker30513
Marker48187
Marker30558
Marker30594
Marker16328
Marker16336
Marker61915
Marker22170
Marker6186 Marker31690
Marker36395
Marker6161
Marker34613
Marker33142 Marker33141
Marker3864
Marker32514
Marker18547
Marker30522
Marker41540
Marker9096
Marker35312
Marker41535
Marker11594
Marker6223
Marker3721 Marker32188
Marker35322
Marker17766
Marker29570
Marker28690
Marker6736
Marker18957
Marker3990
Marker15291
Marker41538
Marker18692
Marker9859
Marker9838
Marker50812 Marker47016
Marker49385 Marker49639
Marker392
Marker10740
Marker43898
Marker6154
Marker10651
Marker6978
Marker6311
Marker24448
Marker34627
Marker34416
Marker40341
Marker33151
Marker37727
Marker19149
Marker51726
Marker6092
Marker16345
Marker16514
Marker61107
Marker43063
Marker17650
Marker33764 Marker3093
Marker31656
Marker42339
Marker32250
Marker3700
Marker10155
Marker39292
Marker36008
Marker19146
Marker35944
Marker25342
Marker3346
Marker34151
Marker35050 Marker15846
Marker40453
Marker40419
Marker33655
Marker43306
Marker43167
Marker16513
Marker12971
Marker8240
Marker7198
Marker18698 Marker27661
Marker38843
Marker3743 Marker18955
Marker28684
Marker15280
Marker40454
Marker9269
Marker4913
Marker4914
Marker40482
Marker20930
Marker35022
Marker763
Marker37247
Marker21346
Marker35021
Marker149
Marker8710
Marker35402
Marker206
Marker31652
Marker20608
Marker15843
Marker32772
Marker3676
Marker43165
Marker7194
Marker36916
Marker18715
Marker18690 Marker18691
Marker40408

LG A2

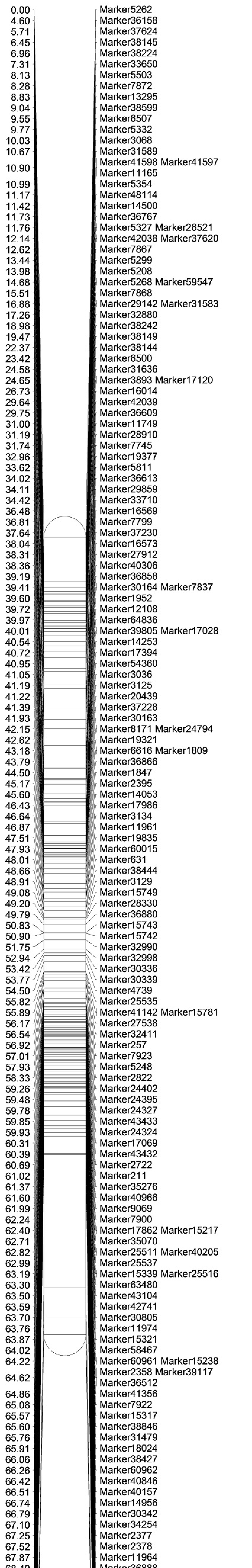
0.00 Marker5262
4.60 Marker36158
5.71 Marker37624
6.45 Marker38145
6.96 Marker38224
7.31 Marker33650
8.13 Marker5503
8.28 Marker7872
8.83 Marker13295
9.04 Marker38599
9.55 Marker6507
9.77 Marker5332
10.03 Marker3068
10.67 Marker31589
10.90 Marker41598 Marker41597
Marker11165
10.99 Marker5354
11.17 Marker48114
11.42 Marker14500
11.73 Marker36767
11.76 Marker5327 Marker26521
12.14 Marker42038 Marker37620
12.62 Marker7867
13.44 Marker5299
13.98 Marker5208
14.68 Marker5268 Marker59547
15.51 Marker7868
16.88 Marker29142 Marker31583
17.26 Marker32880
18.98 Marker38242
19.47 Marker38149
22.37 Marker38144
23.42 Marker6500
24.58 Marker31636
24.65 Marker3893 Marker17120
26.73 Marker16014
29.64 Marker42039
29.75 Marker36609
31.00 Marker11749
31.19 Marker28910
31.74 Marker7745
32.96 Marker19377
33.62 Marker5811
34.02 Marker36613
34.11 Marker29859
34.42 Marker33710
36.48 Marker16569
36.81 Marker7799
37.64 Marker37230
38.04 Marker16573
38.31 Marker27912
38.36 Marker40306
39.19 Marker36858
39.41 Marker30164 Marker7837
39.60 Marker1952
39.72 Marker12108
39.97 Marker64836
40.01 Marker39805 Marker17028
40.54 Marker14253
40.72 Marker17394
40.95 Marker54360
41.05 Marker3036
41.19 Marker3125
41.22 Marker20439
41.39 Marker37228
41.93 Marker30163
42.15 Marker8171 Marker24794
42.62 Marker19321
43.18 Marker6616 Marker1809
43.79 Marker36866
44.50 Marker1847
45.17 Marker2395
45.60 Marker14053
46.43 Marker17986
46.64 Marker3134
46.87 Marker11961
47.51 Marker19835
47.93 Marker60015
48.01 Marker631
48.66 Marker38444
48.91 Marker3129
49.08 Marker15749
49.20 Marker28330
49.79 Marker36880
50.83 Marker15743
50.90 Marker15742
51.75 Marker32990
52.94 Marker32998
53.42 Marker30336
53.77 Marker30339
54.50 Marker4739
55.82 Marker25535
55.89 Marker41142 Marker15781
56.17 Marker27538
56.54 Marker32411
56.92 Marker257
57.01 Marker7923
57.93 Marker5248
58.33 Marker2822
59.26 Marker24402
59.48 Marker24395
59.78 Marker24327
59.85 Marker43433
59.93 Marker24324
60.31 Marker17069
60.39 Marker43432
60.69 Marker2722
61.02 Marker211
61.37 Marker35276
61.60 Marker40966
61.99 Marker9069
62.24 Marker7900
62.40 Marker17862 Marker15217
62.71 Marker35070
62.82 Marker25511 Marker40205
62.99 Marker25537
63.19 Marker15339 Marker25516
63.30 Marker63480
63.50 Marker43104
63.59 Marker42741
63.70 Marker30805
63.76 Marker11974
63.87 Marker15321
64.02 Marker58467
64.22 Marker60961 Marker15238
64.62 Marker2358 Marker39117
Marker36512
64.86 Marker41356
65.08 Marker7922
65.57 Marker15317
65.60 Marker38846
65.76 Marker31479
65.91 Marker18024
66.06 Marker38427
66.26 Marker60962
66.42 Marker40846
66.51 Marker40157
66.74 Marker14956
66.79 Marker30342
67.10 Marker34254
67.25 Marker2377
67.52 Marker2378
67.87 Marker11964
68.40 Marker36888
68.64 Marker32327 Marker19501
69.11 Marker43049
69.42 Marker30691
69.57 Marker33001
69.94 Marker14251
70.02 Marker5203
70.24 Marker11963
70.62 Marker7940
70.86 Marker35764
71.35 Marker5831
71.64 Marker43962
72.45 Marker3043
73.20 Marker33812
74.29 Marker36882
74.96 Marker14084
75.58 Marker40545
75.66 Marker21383
76.03 Marker2650
76.53 Marker18003
76.85 Marker17987
76.98 Marker14080 Marker35060
79.20 Marker7914
79.36 Marker30348
96.48 Marker15747
100.37 Marker26269
102.50 Marker15219
Marker2355

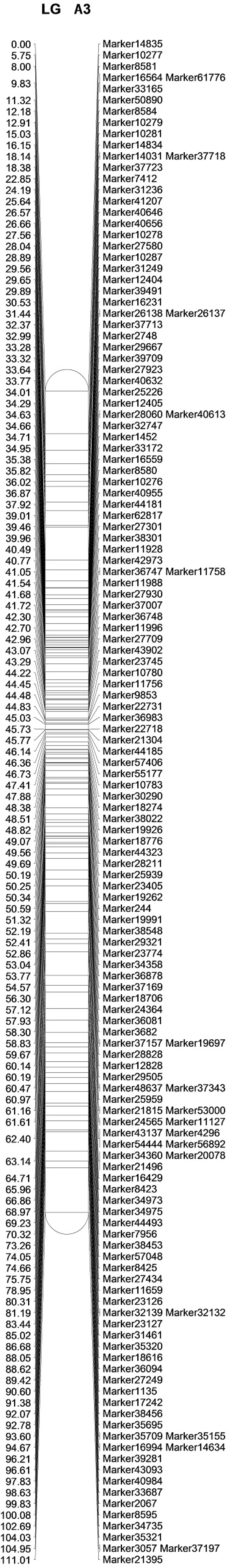
LG A3
0.00
5.75
8.00
9.83
11.32
12.18
12.91
15.03
16.15
18.14
18.38
22.85
24.19
25.64
26.57
26.66
27.56
28.04
28.89
29.56
29.65
29.89
30.53
31.44
32.37
32.99
33.28
33.32
33.64
33.77
34.01
34.29
34.63
34.66
34.71
34.95
35.38
35.82
36.02
36.87
37.92
39.01
39.46
39.96
40.49
40.77
41.05
41.54
41.68
41.72
42.30
42.70
42.96
43.07
43.29
44.22
44.45
44.48
44.83
45.03
45.73
45.77
46.14
46.36
46.73
47.41
47.88
48.38
48.51
48.82
49.07
49.56
49.69
50.19
50.25
50.34
50.59
51.32
52.19
52.41
52.86
53.04
53.77
54.57
56.30
57.12
57.93
58.30
58.83
59.67
60.14
60.19
60.47
60.97
61.16
61.61
62.40
63.14
64.71
65.96
66.86
68.97
69.23
70.32
73.26
74.05
74.66
75.75
78.95
80.31
81.19
83.44
85.02
86.68
88.05
88.62
89.42
90.60
91.38
92.07
92.78
93.60
94.67
96.21
96.61
97.83
98.63
99.83
100.08
102.69
104.03
104.95
111.01
Marker14835
Marker10277
Marker8581
Marker16564 Marker61776
Marker33165
Marker50890
Marker8584
Marker10279
Marker10281
Marker14834
Marker14031 Marker37718
Marker37723
Marker7412
Marker31236
Marker41207
Marker40646
Marker40656
Marker10278
Marker27580
Marker10287
Marker31249
Marker12404
Marker39491
Marker16231
Marker26138 Marker26137
Marker37713
Marker2748
Marker29667
Marker39709
Marker27923
Marker40632
Marker25226
Marker12405
Marker28060 Marker40613
Marker32747
Marker1452
Marker33172
Marker16559
Marker8580
Marker10276
Marker40955
Marker44181
Marker62817
Marker27301
Marker38301
Marker11928
Marker42973
Marker36747 Marker11758
Marker11988
Marker27930
Marker37007
Marker36748
Marker11996
Marker27709
Marker43902
Marker23745
Marker10780
Marker11756
Marker9853
Marker22731
Marker36983
Marker22718
Marker21304
Marker44185
Marker57406
Marker55177
Marker10783
Marker30290
Marker18274
Marker38022
Marker19926
Marker18776
Marker44323
Marker28211
Marker25939
Marker23405
Marker19262
Marker244
Marker19991
Marker38548
Marker29321
Marker23774
Marker34358
Marker36878
Marker37169
Marker18706
Marker24364
Marker36081
Marker3682
Marker37157 Marker19697
Marker28828
Marker12828
Marker29505
Marker48637 Marker37343
Marker25959
Marker21815 Marker53000
Marker24565 Marker11127
Marker43137 Marker4296
Marker54444 Marker56892
Marker34360 Marker20078
Marker21496
Marker16429
Marker8423
Marker34973
Marker34975
Marker44493
Marker7956
Marker38453
Marker57048
Marker8425
Marker27434
Marker11659
Marker23126
Marker32139 Marker32132
Marker23127
Marker31461
Marker35320
Marker18616
Marker36094
Marker27249
Marker1135
Marker17242
Marker38456
Marker35695
Marker35709 Marker35155
Marker16994 Marker14634
Marker39281
Marker43093
Marker40984
Marker33687
Marker2067
Marker8595
Marker34735
Marker35321
Marker3057 Marker37197
Marker21395

LG A4

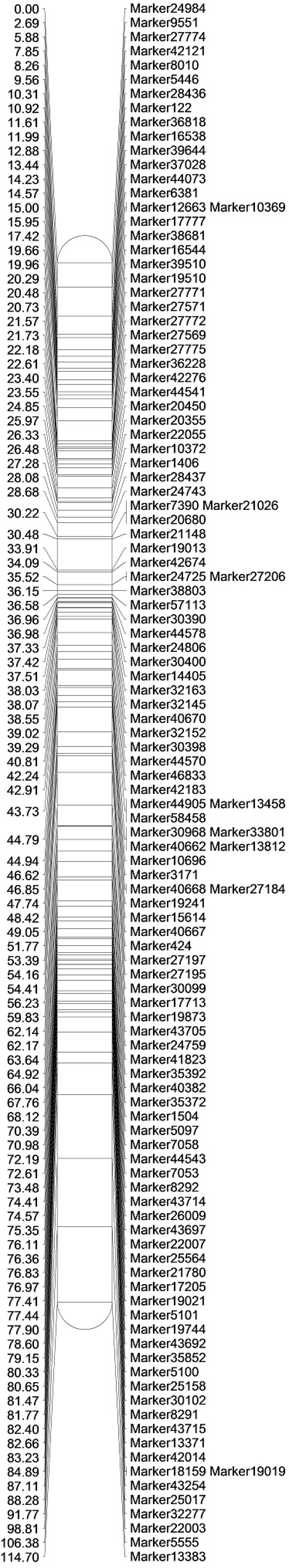
0.00 Marker24984
2.69 Marker9551
5.88 Marker27774
7.85 Marker42121
8.26 Marker8010
9.56 Marker5446
10.31 Marker28436
10.92 Marker122
11.61 Marker36818
11.99 Marker16538
12.88 Marker39644
13.44 Marker37028
14.23 Marker44073
14.57 Marker6381
15.00 Marker12663 Marker10369
15.95 Marker17777
17.42 Marker38681
19.66 Marker16544
19.96 Marker39510
20.29 Marker19510
20.48 Marker27771
20.73 Marker27571
21.57 Marker27772
21.73 Marker27569
22.18 Marker27775
22.61 Marker36228
23.40 Marker42276
23.55 Marker44541
24.85 Marker20450
25.97 Marker20355
26.33 Marker22055
26.48 Marker10372
27.28 Marker1406
28.08 Marker28437
28.68 Marker24743
30.22 Marker7390 Marker21026
Marker20680
30.48 Marker21148
33.91 Marker19013
34.09 Marker42674
35.52 Marker24725 Marker27206
36.15 Marker38803
36.58 Marker57113
36.96 Marker30390
36.98 Marker44578
37.33 Marker24806
37.42 Marker30400
37.51 Marker14405
38.03 Marker32163
38.07 Marker32145
38.55 Marker40670
39.02 Marker32152
39.29 Marker30398
40.81 Marker44570
42.24 Marker46833
42.91 Marker42183
43.73 Marker44905 Marker13458
Marker58458
44.79 Marker30968 Marker33801
Marker40662 Marker13812
44.94 Marker10696
46.62 Marker3171
46.85 Marker40668 Marker27184
47.74 Marker19241
48.42 Marker15614
49.05 Marker40667
51.77 Marker424
53.39 Marker27197
54.16 Marker27195
54.41 Marker30099
56.23 Marker17713
59.83 Marker19873
62.14 Marker43705
62.17 Marker24759
63.64 Marker41823
64.92 Marker35392
66.04 Marker40382
67.76 Marker35372
68.12 Marker1504
70.39 Marker5097
70.98 Marker7058
72.19 Marker44543
72.61 Marker7053
73.48 Marker8292
74.41 Marker43714
74.57 Marker26009
75.35 Marker43697
76.11 Marker22007
76.36 Marker25564
76.83 Marker21780
76.97 Marker17205
77.41 Marker19021
77.44 Marker5101
77.90 Marker19744
78.60 Marker43692
79.15 Marker35852
80.33 Marker5100
80.65 Marker25158
81.47 Marker30102
81.77 Marker8291
82.40 Marker43715
82.66 Marker13371
83.23 Marker42014
84.89 Marker18159 Marker19019
87.11 Marker43254
88.28 Marker25017
91.77 Marker32277
98.81 Marker22003
106.38 Marker5555
114.70 Marker13383

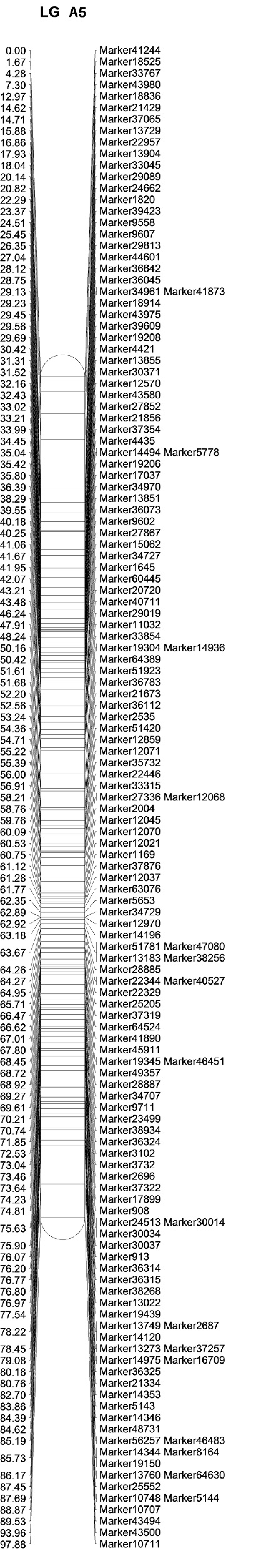
LG A5
0.00 Marker41244
1.67 Marker18525
4.28 Marker33767
7.30 Marker43980
12.97 Marker18836
14.62 Marker21429
14.71 Marker37065
15.88 Marker13729
16.86 Marker22957
17.93 Marker13904
18.04 Marker33045
20.14 Marker29089
20.82 Marker24662
22.29 Marker1820
23.37 Marker39423
24.51 Marker9558
25.45 Marker9607
26.35 Marker29813
27.04 Marker44601
28.12 Marker36642
28.75 Marker36045
29.13 Marker34961 Marker41873
29.23 Marker18914
29.45 Marker43975
29.56 Marker39609
29.69 Marker19208
30.42 Marker4421
31.31 Marker13855
31.52 Marker30371
32.16 Marker12570
32.43 Marker43580
33.02 Marker27852
33.21 Marker21856
33.99 Marker37354
34.45 Marker4435
35.04 Marker14494 Marker5778
35.42 Marker19206
35.80 Marker17037
36.39 Marker34970
38.29 Marker13851
39.55 Marker36073
40.18 Marker9602
40.25 Marker27867
41.06 Marker15062
41.67 Marker34727
41.95 Marker1645
42.07 Marker60445
43.21 Marker20720
43.48 Marker40711
46.24 Marker29019
47.91 Marker11032
48.24 Marker33854
50.16 Marker19304 Marker14936
50.42 Marker64389
51.61 Marker51923
51.68 Marker36783
52.20 Marker21673
52.56 Marker36112
53.24 Marker2535
54.36 Marker51420
54.71 Marker12859
55.22 Marker12071
55.39 Marker35732
56.00 Marker22446
56.91 Marker33315
58.21 Marker27336 Marker12068
58.76 Marker2004
59.76 Marker12045
60.09 Marker12070
60.53 Marker12021
60.75 Marker1169
61.12 Marker37876
61.28 Marker12037
61.77 Marker63076
62.35 Marker5653
62.89 Marker34729
62.92 Marker12970
63.18 Marker14196
63.67 Marker51781 Marker47080
Marker13183 Marker38256
64.26 Marker28885
64.27 Marker22344 Marker40527
64.95 Marker22329
65.71 Marker25205
66.47 Marker37319
66.62 Marker64524
67.01 Marker41890
67.80 Marker45911
68.45 Marker19345 Marker46451
68.72 Marker49357
68.92 Marker28887
69.27 Marker34707
69.61 Marker9711
70.21 Marker23499
70.74 Marker38934
71.85 Marker36324
72.53 Marker3102
73.04 Marker3732
73.46 Marker2696
73.64 Marker37322
74.23 Marker17899
74.81 Marker908
75.63 Marker24513 Marker30014
Marker30034
75.90 Marker30037
76.07 Marker913
76.20 Marker36314
76.77 Marker36315
76.80 Marker38268
76.97 Marker13022
77.54 Marker19439
78.22 Marker13749 Marker2687
Marker14120
78.45 Marker13273 Marker37257
79.08 Marker14975 Marker16709
80.18 Marker36325
80.76 Marker21334
82.70 Marker14353
83.86 Marker5143
84.39 Marker14346
84.62 Marker48731
85.19 Marker56257 Marker46483
85.73 Marker14344 Marker8164
Marker19150
86.17 Marker13760 Marker64630
87.45 Marker25552
87.69 Marker10748 Marker5144
88.87 Marker10707
89.53 Marker43494
93.96 Marker43500
97.88 Marker10711

LG A6

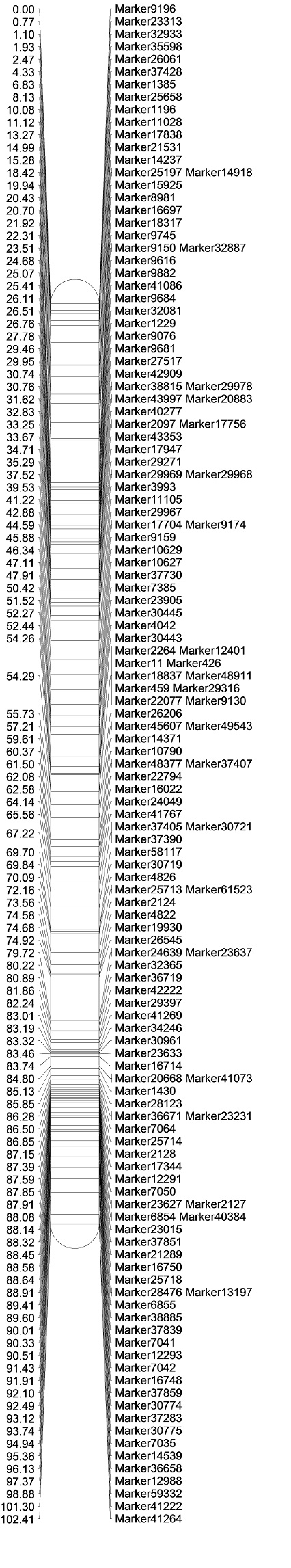
0.00 Marker9196
0.77 Marker23313
1.10 Marker32933
1.93 Marker35598
2.47 Marker26061
4.33 Marker37428
6.83 Marker1385
8.13 Marker25658
10.08 Marker1196
11.12 Marker11028
13.27 Marker17838
14.99 Marker21531
15.28 Marker14237
18.42 Marker25197 Marker14918
19.94 Marker15925
20.43 Marker8981
20.70 Marker16697
21.92 Marker18317
22.31 Marker9745
23.51 Marker9150 Marker32887
24.68 Marker9616
25.07 Marker9882
25.41 Marker41086
26.11 Marker9684
26.51 Marker32081
26.76 Marker1229
27.78 Marker9076
29.46 Marker9681
29.95 Marker27517
30.74 Marker42909
30.76 Marker38815 Marker29978
31.62 Marker43997 Marker20883
32.83 Marker40277
33.25 Marker2097 Marker17756
33.67 Marker43353
34.71 Marker17947
35.29 Marker29271
37.52 Marker29969 Marker29968
39.53 Marker3993
41.22 Marker11105
42.88 Marker29967
44.59 Marker17704 Marker9174
45.88 Marker9159
46.34 Marker10629
47.11 Marker10627
47.91 Marker37730
50.42 Marker7385
51.52 Marker23905
52.27 Marker30445
52.44 Marker4042
54.26 Marker30443
Marker2264 Marker12401
Marker11 Marker426
54.29 Marker18837 Marker48911
Marker459 Marker29316
Marker22077 Marker9130
55.73 Marker26206
57.21 Marker45607 Marker49543
59.61 Marker14371
60.37 Marker10790
61.50 Marker48377 Marker37407
62.08 Marker22794
62.58 Marker16022
64.14 Marker24049
65.56 Marker41767
Marker37405 Marker30721
67.22
Marker37390
69.70 Marker58117
69.84 Marker30719
70.09 Marker4826
72.16 Marker25713 Marker61523
73.56 Marker2124
74.58 Marker4822
74.68 Marker19930
74.92 Marker26545
79.72 Marker24639 Marker23637
80.22 Marker32365
80.89 Marker36719
81.86 Marker42222
82.24 Marker29397
83.01 Marker41269
83.19 Marker34246
83.32 Marker30961
83.46 Marker23633
83.74 Marker16714
84.80 Marker20668 Marker41073
85.13 Marker1430
85.85 Marker28123
86.28 Marker36671 Marker23231
86.50 Marker7064
86.85 Marker25714
87.15 Marker2128
87.39 Marker17344
87.59 Marker12291
87.85 Marker7050
87.91 Marker23627 Marker2127
88.08 Marker6854 Marker40384
88.14 Marker23015
88.32 Marker37851
88.45 Marker21289
88.58 Marker16750
88.64 Marker25718
88.91 Marker28476 Marker13197
89.41 Marker6855
89.60 Marker38885
90.01 Marker37839
90.33 Marker7041
90.51 Marker12293
91.43 Marker7042
91.91 Marker16748
92.10 Marker37859
92.49 Marker30774
93.12 Marker37283
93.74 Marker30775
94.94 Marker7035
95.36 Marker14539
96.13 Marker36658
97.37 Marker12988
98.88 Marker59332
101.30 Marker41222
102.41 Marker41264

LG A7

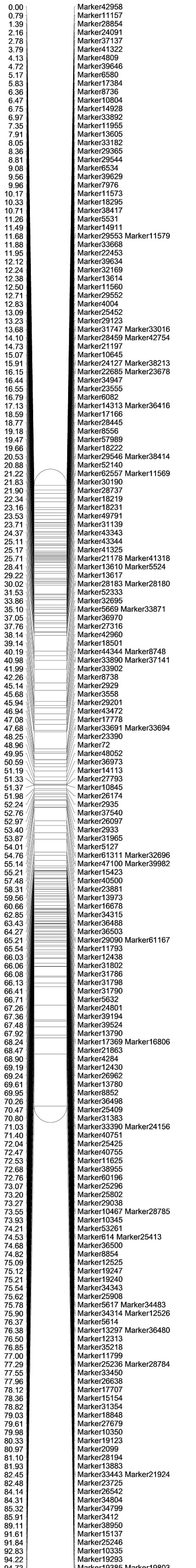
0.00 Marker42958
0.79 Marker11157
1.39 Marker28854
2.16 Marker24091
2.78 Marker37137
3.79 Marker41322
4.13 Marker4809
4.72 Marker39646
5.17 Marker6580
5.83 Marker17384
6.36 Marker8736
6.47 Marker10804
6.75 Marker14928
6.97 Marker33892
7.35 Marker11955
7.91 Marker13605
8.05 Marker33182
8.36 Marker29365
8.81 Marker29544
9.08 Marker6534
9.56 Marker39629
9.96 Marker7976
10.17 Marker11573
10.33 Marker18295
10.71 Marker38417
11.26 Marker5531
11.49 Marker14911
11.68 Marker29553 Marker11579
11.88 Marker33668
11.95 Marker22453
12.12 Marker39634
12.24 Marker32169
12.38 Marker13614
12.50 Marker11560
12.71 Marker29552
12.83 Marker4004
13.09 Marker25452
13.23 Marker29123
13.68 Marker31747 Marker33016
14.10 Marker28459 Marker42754
14.73 Marker21197
15.07 Marker10645
15.91 Marker24127 Marker38213
16.15 Marker22685 Marker23678
16.44 Marker34947
16.55 Marker23555
16.79 Marker6082
17.13 Marker14313 Marker36416
18.59 Marker17166
18.77 Marker28445
19.18 Marker8556
19.47 Marker57989
19.66 Marker18222
20.53 Marker29546 Marker38414
20.88 Marker52140
21.22 Marker62557 Marker11569
21.83 Marker30190
21.90 Marker28737
22.34 Marker18219
23.16 Marker18231
23.53 Marker49791
23.71 Marker31139
24.37 Marker43343
25.11 Marker43344
25.17 Marker41325
25.71 Marker21178 Marker41318
28.41 Marker13610 Marker5524
29.22 Marker13617
30.02 Marker28183 Marker28180
31.53 Marker52333
33.86 Marker32695
35.10 Marker5669 Marker33871
37.05 Marker36970
37.76 Marker27316
38.14 Marker42960
39.14 Marker18501
40.19 Marker44344 Marker8748
40.98 Marker33890 Marker37141
41.99 Marker33902
42.26 Marker8738
45.14 Marker2929
45.68 Marker3558
45.94 Marker29201
46.94 Marker43472
47.08 Marker17778
47.68 Marker33691 Marker33694
48.25 Marker23390
48.96 Marker72
49.95 Marker48052
50.59 Marker36973
51.19 Marker14113
51.33 Marker27793
51.37 Marker10845
51.98 Marker26174
52.24 Marker2935
52.76 Marker37540
52.97 Marker26097
53.40 Marker2933
53.87 Marker31965
54.01 Marker5127
54.76 Marker61311 Marker32696
55.14 Marker47100 Marker39982
55.21 Marker15423
57.48 Marker40500
58.31 Marker23881
59.56 Marker13973
60.66 Marker16678
62.85 Marker34315
63.43 Marker36488
64.27 Marker36503
65.21 Marker29090 Marker61167
65.54 Marker11793
66.03 Marker12438
66.06 Marker31802
66.08 Marker31786
66.13 Marker31798
66.41 Marker31790
66.71 Marker5632
67.26 Marker24801
67.36 Marker39194
67.48 Marker39524
67.92 Marker13790
68.24 Marker17369 Marker16806
68.47 Marker21863
68.90 Marker4284
69.19 Marker12430
69.24 Marker26962
69.61 Marker13780
69.95 Marker8852
70.26 Marker36498
70.47 Marker25409
70.80 Marker31383
71.03 Marker33390 Marker24156
71.40 Marker40751
72.04 Marker25425
72.47 Marker40755
72.53 Marker11625
72.68 Marker38955
72.76 Marker60196
73.07 Marker25296
73.20 Marker25802
73.27 Marker29038
73.55 Marker10467 Marker28785
73.93 Marker10345
74.21 Marker53261
74.53 Marker614 Marker25413
74.68 Marker36500
74.82 Marker8854
75.09 Marker12525
75.12 Marker19247
75.21 Marker19240
75.54 Marker34343
75.62 Marker25908
75.78 Marker5617 Marker34483
75.90 Marker34314 Marker12526
76.37 Marker5614
76.38 Marker13297 Marker36480
76.50 Marker12313
76.85 Marker35218
77.00 Marker11799
77.29 Marker25236 Marker28784
77.55 Marker33450
77.96 Marker26638
78.12 Marker17707
78.36 Marker15154
78.82 Marker31354
79.03 Marker18848
79.61 Marker27679
79.98 Marker10350
80.33 Marker19123
80.97 Marker2099
81.10 Marker28194
81.93 Marker13883
82.45 Marker33443 Marker21924
82.48 Marker23725
84.14 Marker26542
84.31 Marker34804
85.32 Marker34799
85.91 Marker3412
89.11 Marker38950
91.61 Marker15137
91.84 Marker25246
92.83 Marker10335
94.22 Marker19293
94.73 Marker19385 Marker19803
97.47 Marker31960
106.41 Marker13841

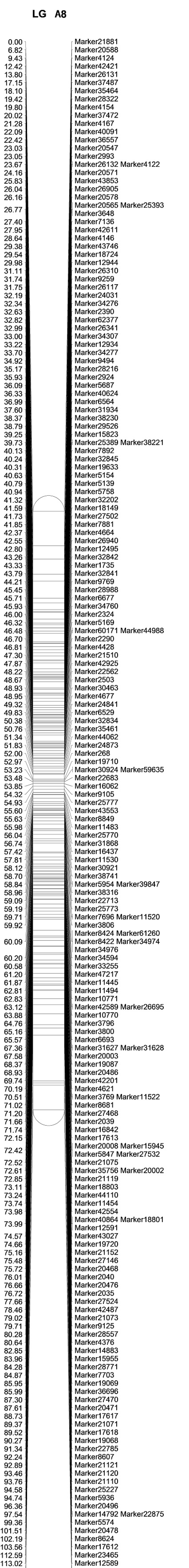
LG A8
0.00 Marker21881
6.82 Marker20588
9.43 Marker4124
12.42 Marker42421
13.80 Marker26131
17.15 Marker37487
18.10 Marker35464
19.42 Marker28322
19.80 Marker4154
20.02 Marker37472
21.28 Marker4167
22.09 Marker40091
22.42 Marker36557
23.03 Marker20547
23.05 Marker2993
23.67 Marker26132 Marker4122
24.16 Marker20571
25.83 Marker43853
26.04 Marker26905
26.16 Marker20578
26.77 Marker20565 Marker25393 Marker3648
27.40 Marker7136
27.95 Marker42611
28.64 Marker4146
29.38 Marker43746
29.54 Marker18724
29.98 Marker12944
31.11 Marker26310
31.74 Marker9259
31.75 Marker26117
32.19 Marker24031
32.34 Marker34276
32.63 Marker2390
32.82 Marker62377
32.99 Marker26341
33.00 Marker34307
33.22 Marker12934
33.70 Marker34277
34.92 Marker9494
35.17 Marker28216
35.93 Marker2924
36.09 Marker5687
36.33 Marker40624
36.99 Marker6564
37.60 Marker31934
38.37 Marker38230
38.79 Marker29526
39.25 Marker15823
39.73 Marker25389 Marker38221
40.13 Marker7892
40.24 Marker32845
40.31 Marker19633
40.63 Marker5154
40.79 Marker5139
40.94 Marker5758
41.32 Marker32202
41.59 Marker18149
41.73 Marker27502
41.85 Marker7881
42.37 Marker4664
42.55 Marker26940
42.80 Marker12495
43.26 Marker32842
43.33 Marker1735
43.79 Marker32841
44.21 Marker9769
45.45 Marker28988
45.71 Marker6677
45.93 Marker34760
46.00 Marker2324
46.32 Marker5169
46.48 Marker60171 Marker44988
46.70 Marker2290
46.81 Marker4428
47.30 Marker21510
47.87 Marker42925
48.22 Marker22562
48.67 Marker2503
48.93 Marker30463
48.95 Marker4677
49.32 Marker24841
49.83 Marker6529
50.38 Marker32834
50.76 Marker35461
51.34 Marker44062
51.83 Marker24873
52.00 Marker268
52.97 Marker19710
53.23 Marker30924 Marker59635
53.48 Marker22683
53.85 Marker16062
54.32 Marker9105
54.93 Marker25777
55.60 Marker43553
55.63 Marker8849
55.98 Marker11483
56.04 Marker25770
56.74 Marker31868
57.42 Marker16437
57.81 Marker11530
58.12 Marker30921
58.70 Marker38741
58.84 Marker5954 Marker39847
58.96 Marker38316
59.09 Marker22713
59.19 Marker25773
59.71 Marker7696 Marker11520
59.92 Marker3806
60.09 Marker8424 Marker61260 Marker8422 Marker34974 Marker34976
60.20 Marker34594
60.58 Marker33255
61.20 Marker47217
61.87 Marker11445
62.81 Marker11494
62.83 Marker10771
63.12 Marker42589 Marker26695
63.88 Marker10770
64.76 Marker3796
65.16 Marker3800
65.57 Marker6693
67.36 Marker31627 Marker31628
67.58 Marker20003
68.37 Marker19087
68.93 Marker20486
69.74 Marker42201
70.19 Marker4621
70.51 Marker3769 Marker11522
71.02 Marker8681
71.20 Marker27468
71.66 Marker2039
71.74 Marker16842
72.15 Marker17613
72.42 Marker20008 Marker15945 Marker5847 Marker27532
72.52 Marker21075
72.61 Marker35756 Marker20002
72.85 Marker21119
73.11 Marker18803
73.24 Marker44110
73.74 Marker11454
73.98 Marker42554
73.99 Marker40864 Marker18801 Marker12591
74.57 Marker43027
74.66 Marker19720
75.16 Marker21152
75.48 Marker27146
75.72 Marker20468
76.01 Marker2040
76.66 Marker20476
76.72 Marker2035
77.66 Marker27524
78.46 Marker42487
79.02 Marker21073
79.71 Marker9125
80.28 Marker28557
80.64 Marker4376
82.85 Marker14883
83.96 Marker15955
84.28 Marker28771
84.87 Marker7703
85.95 Marker19069
85.99 Marker36696
87.30 Marker27470
87.61 Marker20471
88.73 Marker17617
89.37 Marker21071
89.52 Marker17618
90.27 Marker19068
91.34 Marker22785
92.24 Marker8607
92.89 Marker21121
93.46 Marker21120
93.76 Marker21110
94.58 Marker25227
94.74 Marker5936
96.36 Marker20496
97.54 Marker14792 Marker22875
99.36 Marker5574
101.51 Marker20478
102.19 Marker8624
103.56 Marker17612
112.59 Marker23465
113.02 Marker12589
113.46 Marker270
118.35 Marker27481

LG A9

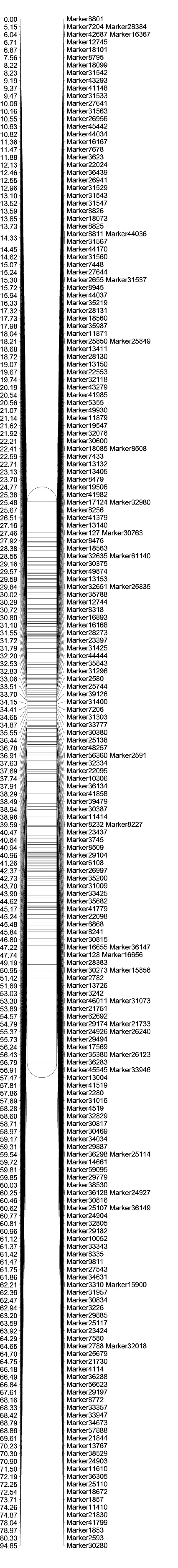

0.00 Marker8801
5.15 Marker7204 Marker28384
6.04 Marker42687 Marker16367
6.71 Marker12745
6.87 Marker18101
7.56 Marker8795
8.22 Marker18099
8.23 Marker31542
9.19 Marker43293
9.37 Marker41148
9.47 Marker31533
10.06 Marker27641
10.16 Marker31563
10.55 Marker26956
10.63 Marker45442
10.82 Marker44034
11.36 Marker16167
11.47 Marker7678
11.88 Marker3623
12.13 Marker22024
12.46 Marker36439
12.55 Marker26941
12.96 Marker31529
13.10 Marker31543
13.52 Marker31547
13.59 Marker8826
13.65 Marker18073
13.73 Marker8825
14.33 Marker8811 Marker44036 Marker31567
14.45 Marker44170
14.62 Marker31560
15.07 Marker7448
15.24 Marker27644
15.30 Marker2655 Marker31537
15.72 Marker8945
15.94 Marker44037
16.33 Marker35219
17.32 Marker28131
17.73 Marker18560
17.98 Marker35987
18.04 Marker11871
18.21 Marker25850 Marker25849
18.68 Marker13411
18.72 Marker28130
19.07 Marker13150
19.67 Marker22553
19.74 Marker32118
20.19 Marker43279
20.54 Marker41985
20.56 Marker5355
21.07 Marker49930
21.14 Marker11879
21.62 Marker19547
21.92 Marker32076
22.21 Marker30600
22.41 Marker18085 Marker8508
22.59 Marker7433
22.71 Marker13132
23.13 Marker13405
23.70 Marker8479
24.77 Marker19506
25.38 Marker41982
25.48 Marker17124 Marker32980
25.67 Marker8256
26.51 Marker41379
27.16 Marker13140
27.46 Marker127 Marker30763
27.92 Marker8476
28.38 Marker18563
28.55 Marker32635 Marker61140
29.16 Marker30375
29.57 Marker49874
29.59 Marker13153
29.84 Marker32651 Marker25835
30.02 Marker35788
30.29 Marker12744
30.72 Marker8318
30.80 Marker16893
31.10 Marker16168
31.55 Marker28273
31.72 Marker23397
31.79 Marker31425
32.20 Marker44444
32.53 Marker35843
32.83 Marker31296
33.06 Marker2580
33.51 Marker25744
33.70 Marker39126
34.15 Marker31400
34.41 Marker7206
34.65 Marker31303
34.87 Marker33777
35.55 Marker30380
36.44 Marker25138
36.78 Marker48257
36.91 Marker56360 Marker2591
37.63 Marker32334
37.69 Marker22095
37.74 Marker10306
37.91 Marker36134
38.29 Marker41858
38.49 Marker39479
38.94 Marker30387
38.98 Marker11414
39.59 Marker8232 Marker8227
40.47 Marker23437
40.64 Marker3745
40.94 Marker8509
40.96 Marker29104
41.26 Marker6108
42.37 Marker26997
42.73 Marker35200
43.70 Marker31009
43.90 Marker33425
44.62 Marker35682
45.17 Marker41779
45.24 Marker22098
45.48 Marker6868
45.84 Marker8241
46.80 Marker30815
47.22 Marker16655 Marker36147
47.74 Marker128 Marker16656
49.19 Marker28383
50.95 Marker30273 Marker15856
51.42 Marker2782
51.89 Marker13726
53.03 Marker3242
53.30 Marker46011 Marker31073
53.89 Marker21751
54.57 Marker62692
54.79 Marker29174 Marker21733
55.37 Marker24926 Marker26240
55.73 Marker29494
56.24 Marker17569
56.43 Marker35380 Marker26123
56.79 Marker36283
56.91 Marker45545 Marker33946
57.47 Marker13004
57.81 Marker41519
57.86 Marker2280
57.89 Marker31016
58.28 Marker4519
58.60 Marker32829
58.71 Marker30817
58.97 Marker30469
59.17 Marker34034
59.31 Marker29887
59.54 Marker36298 Marker25114
59.72 Marker14661
59.81 Marker59095
59.85 Marker29779
60.03 Marker38530
60.25 Marker36128 Marker24927
60.46 Marker30816
60.62 Marker25107 Marker36149
60.77 Marker24904
60.81 Marker32805
60.96 Marker29182
61.12 Marker10052
61.37 Marker33343
61.42 Marker8335
61.47 Marker9811
61.75 Marker27543
61.86 Marker34631
62.21 Marker3310 Marker15900
62.36 Marker31957
62.47 Marker30834
62.94 Marker3226
63.20 Marker29885
63.59 Marker25117
63.92 Marker23424
64.29 Marker7580
64.65 Marker2788 Marker32018
64.70 Marker25679
64.75 Marker21730
66.18 Marker4114
66.49 Marker36288
66.84 Marker56623
67.61 Marker29197
68.16 Marker8772
68.33 Marker33357
68.42 Marker33947
68.79 Marker34673
68.86 Marker57888
69.61 Marker21844
70.23 Marker13767
70.30 Marker38529
70.90 Marker24903
71.50 Marker11610
72.19 Marker36305
72.25 Marker25110
72.54 Marker18672
73.71 Marker1857
74.26 Marker11410
74.87 Marker21830
78.04 Marker41799
78.97 Marker1853
80.33 Marker2593
94.65 Marker30280

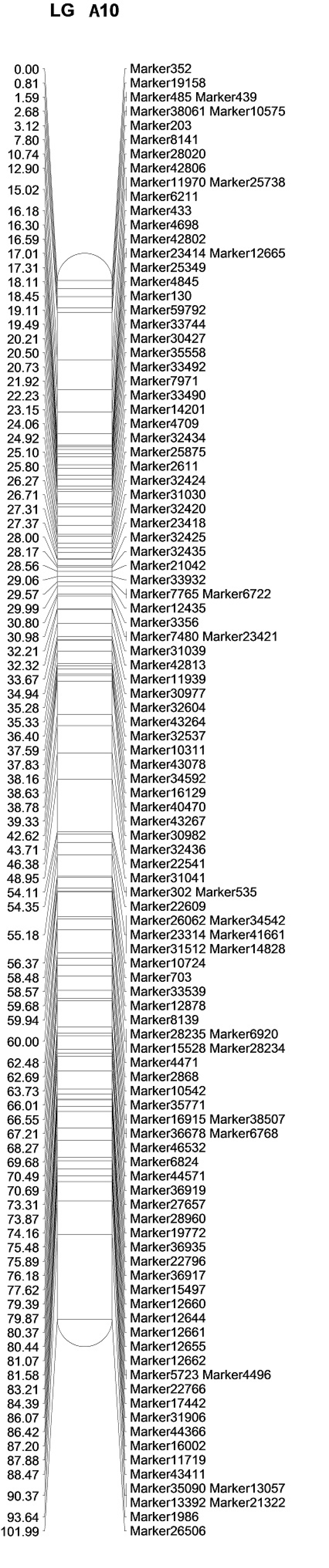
LG A10
0.00
0.81
1.59
2.68
3.12
7.80
10.74
12.90
15.02
16.18
16.30
16.59
17.01
17.31
18.11
18.45
19.11
19.49
20.21
20.50
20.73
21.92
22.23
23.15
24.06
24.92
25.10
25.80
26.27
26.71
27.31
27.37
28.00
28.17
28.56
29.06
29.57
29.99
30.80
30.98
32.21
32.32
33.67
34.94
35.28
35.33
36.40
37.59
37.83
38.16
38.63
38.78
39.33
42.62
43.71
46.38
48.95
54.11
54.35
55.18
56.37
58.48
58.57
59.68
59.94
60.00
62.48
62.69
63.73
66.01
66.55
67.21
68.27
69.68
70.49
70.69
73.31
73.87
74.16
75.48
75.89
76.18
77.62
79.39
79.87
80.37
80.44
81.07
81.58
83.21
84.39
86.07
86.42
87.20
87.88
88.47
90.37
93.64
101.99
Marker352
Marker19158
Marker485 Marker439
Marker38061 Marker10575
Marker203
Marker8141
Marker28020
Marker42806
Marker11970 Marker25738
Marker6211
Marker433
Marker4698
Marker42802
Marker23414 Marker12665
Marker25349
Marker4845
Marker130
Marker59792
Marker33744
Marker30427
Marker35558
Marker33492
Marker7971
Marker33490
Marker14201
Marker4709
Marker32434
Marker25875
Marker2611
Marker32424
Marker31030
Marker32420
Marker23418
Marker32425
Marker32435
Marker21042
Marker33932
Marker7765 Marker6722
Marker12435
Marker3356
Marker7480 Marker23421
Marker31039
Marker42813
Marker11939
Marker30977
Marker32604
Marker43264
Marker32537
Marker10311
Marker43078
Marker34592
Marker16129
Marker40470
Marker43267
Marker30982
Marker32436
Marker22541
Marker31041
Marker302 Marker535
Marker22609
Marker26062 Marker34542
Marker23314 Marker41661
Marker31512 Marker14828
Marker10724
Marker703
Marker33539
Marker12878
Marker8139
Marker28235 Marker6920
Marker15528 Marker28234
Marker4471
Marker2868
Marker10542
Marker35771
Marker16915 Marker38507
Marker36678 Marker6768
Marker46532
Marker6824
Marker44571
Marker36919
Marker27657
Marker28960
Marker19772
Marker36935
Marker22796
Marker36917
Marker15497
Marker12660
Marker12644
Marker12661
Marker12655
Marker12662
Marker5723 Marker4496
Marker22766
Marker17442
Marker31906
Marker44366
Marker16002
Marker11719
Marker43411
Marker35090 Marker13057
Marker13392 Marker21322
Marker1986
Marker26506
