# Supplementary material for: A High-Density SNP Genetic Linkage Map and QTL Analysis of Growth-Related Traits in a Hybrid Family of Oysters (Crassostrea gigas × Crassostrea angulata) Using Genotyping-by-Sequencing
Source: G3 (Bethesda). 2016 Mar 17;6(5):1417–26. doi: 10.1534/g3.116.026971 (PMC4856092; doi:10.1534/g3.116.026971)

Segregation distortion of markers on LG A1

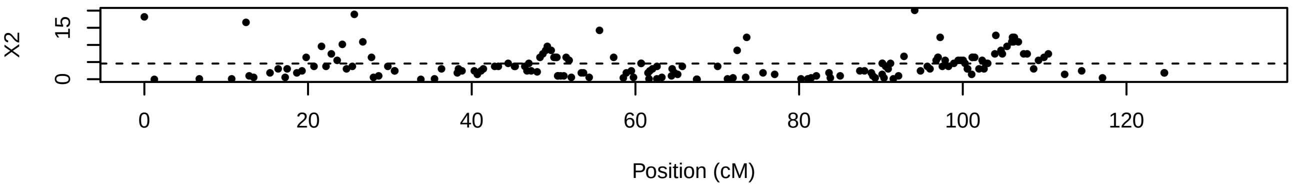

Segregation distortion of markers on LG A2

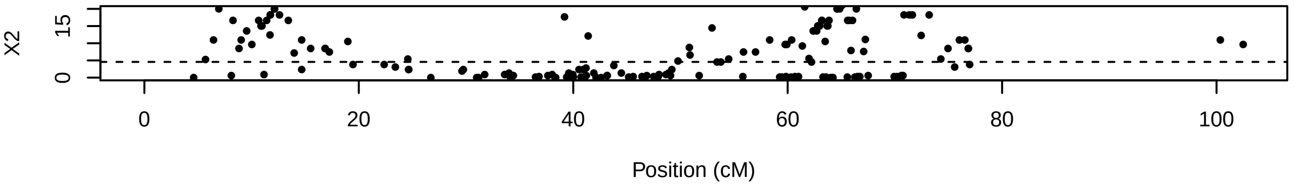

Segregation distortion of markers on LG A3

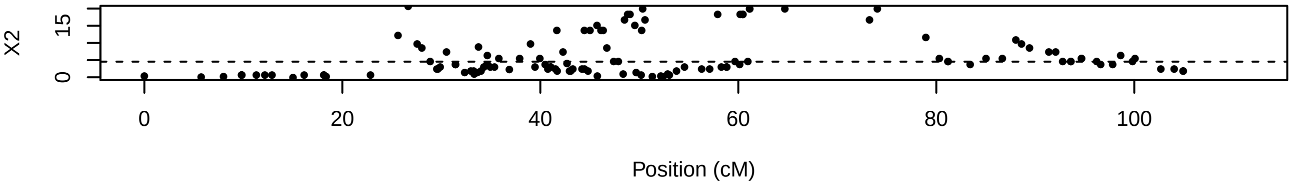

Segregation distortion of markers on LG A4

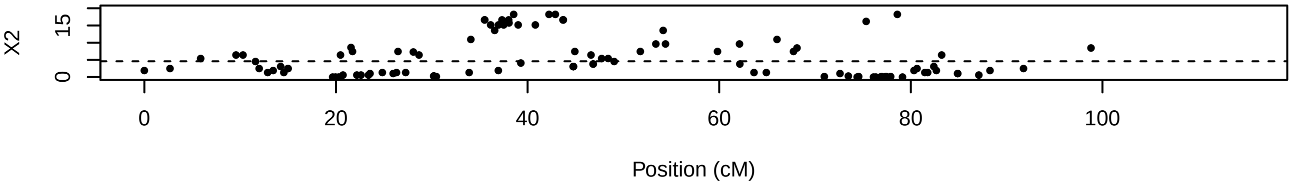

Segregation distortion of markers on LG A5

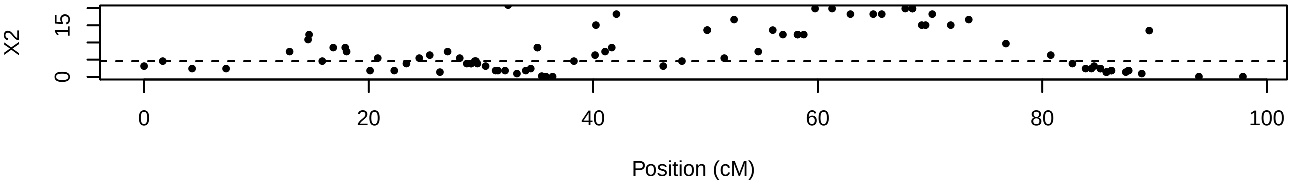

Segregation distortion of markers on LG A6

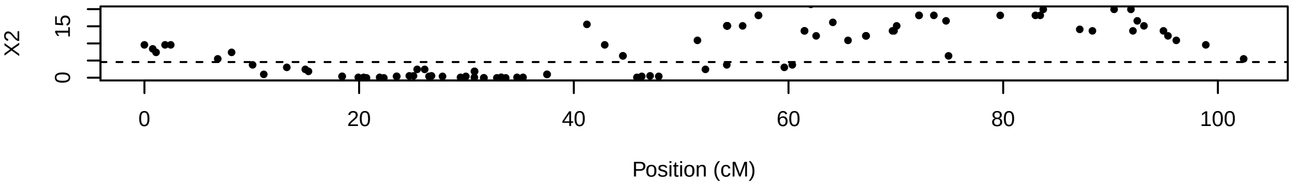

Segregation distortion of markers on LG A7

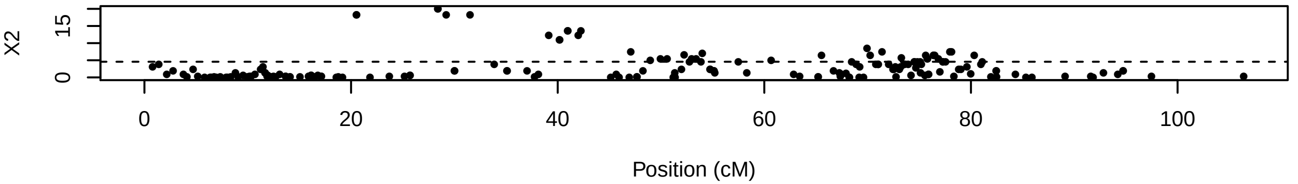

Segregation distortion of markers on LG A8

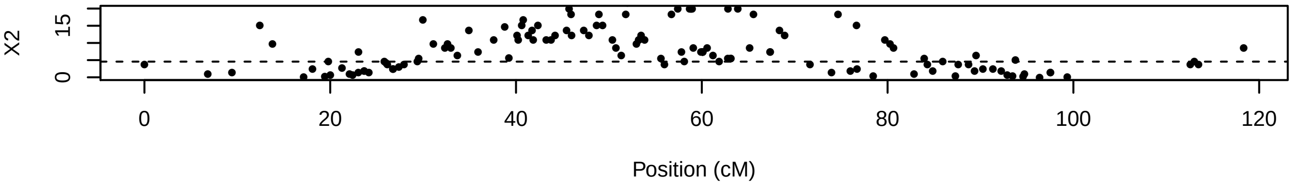

Segregation distortion of markers on LG A9

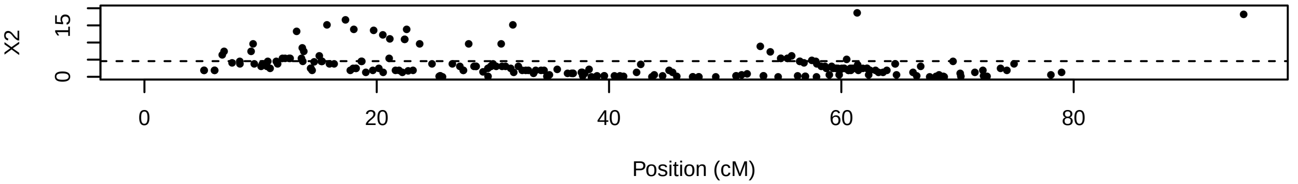

Segregation distortion of markers on LG A10

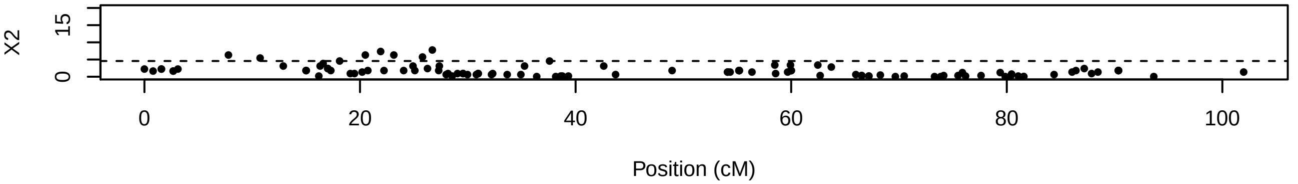

Supplement: Supplemental Material [file supp_g3.116.026971_FileS4.pdf]
